# A Comprehensive Analysis of the Importance of Translation Initiation Factors for *Haloferax volcanii* Applying Deletion and Conditional Depletion Mutants

**DOI:** 10.1371/journal.pone.0077188

**Published:** 2013-11-14

**Authors:** Katrin Gäbel, Jessica Schmitt, Sebastian Schulz, Daniela J. Näther, Jörg Soppa

**Affiliations:** Institute for Molecular Biosciences, Biocentre, Goethe-University, Frankfurt, Germany; The John Curtin School of Medical Research, Australia

## Abstract

Translation is an important step in gene expression. The initiation of translation is phylogenetically diverse, since currently five different initiation mechanisms are known. For bacteria the three initiation factors IF1 – IF3 are described in contrast to archaea and eukaryotes, which contain a considerably higher number of initiation factor genes. As eukaryotes and archaea use a non-overlapping set of initiation mechanisms, orthologous proteins of both domains do not necessarily fulfill the same function. The genome of *Haloferax volcanii* contains 14 annotated genes that encode (subunits of) initiation factors. To gain a comprehensive overview of the importance of these genes, it was attempted to construct single gene deletion mutants of all genes. In 9 cases single deletion mutants were successfully constructed, showing that the respective genes are not essential. In contrast, the genes encoding initiation factors aIF1, aIF2γ, aIF5A, aIF5B, and aIF6 were found to be essential. Factors aIF1A and aIF2β are encoded by two orthologous genes in *H. volcanii*. Attempts to generate double mutants failed in both cases, indicating that also these factors are essential. A translatome analysis of one of the single aIF2β deletion mutants revealed that the translational efficiency of the second ortholog was enhanced tenfold and thus the two proteins can replace one another. The phenotypes of the single deletion mutants also revealed that the two aIF1As and aIF2βs have redundant but not identical functions. Remarkably, the gene encoding aIF2α, a subunit of aIF2 involved in initiator tRNA binding, could be deleted. However, the mutant had a severe growth defect under all tested conditions. Conditional depletion mutants were generated for the five essential genes. The phenotypes of deletion mutants and conditional depletion mutants were compared to that of the wild-type under various conditions, and growth characteristics are discussed.

## Introduction

Translation is a very important step in the expression of genetic information in all three domains of life. It is subdivided into four steps: initiation, elongation, termination and ribosome recycling. Translation initiation is the rate-limiting step of translation and consequently translational regulation often occurs during initiation. Of all four steps translation initiation has the biggest evolutionary divergence, and several different mechanisms co-exist [1], which are shortly described below. Several recent reviews summarize the initiation mechanisms found in bacteria, eukaryotes, and archaea [2]–[11].

The first mechanism involves the so-called Shine Dalgarno (SD) sequence, a motif of 4–8 nt that is localized in the 5′-UTR of transcripts 5–7 nt upstream of the start codon. It interacts via base-pairing with the 3′-end of the 16S rRNA of the small ribosomal subunit and thereby determines the localization of the small subunit on the transcript. This mechanism has been thoroughly characterized in *Escherichia coli* and operates in many bacteria and some archaea. However, it is not the universal mechanism in prokaryotes, in contrast to the view of many publications and text books. A bioinformatic study of 162 available prokaryotic genomes showed that the fraction of genes preceded by a SD sequence ranges from about 15% to more than 90%, dependent on the phylogenetic group [12]. For example, in *Firmicutes* most genes are preceded by a SD sequence, while SD sequences are seldom in *Batceriodetes*. In archaea, in 16 of 21 species less than 50% of genes are preceded by SD sequences, revealing that alternative translation initiation mechanisms predominate in the archaea [12]. Furthermore, a mutational analysis indicated that the SD mechanism does not operate at all in the species used in this study, *Haloferax volcanii* (Kramer et al., submitted).

A second initiation mechanism acts on transcripts lacking 5′-UTRs, which are called leaderless transcripts. It has been proposed that this mechanism might be the evolutionary oldest initiation mechanism, because leaderless transcripts occur in all three domains of life. In addition, the majority of transcripts are leaderless in some species of archaea and thus initiation on leaderless transcripts represents the default mechanism in these species [13]–[16]. Even more pronounced, in the lower eukaryote *Giardia lamblia* all transcripts are leaderless [17], [18]. A bioinformatic survey of 853 bacterial genomes indicated that leaderless transcripts are also widespread in many bacteria, albeit they do not form the predominant fraction [19]. A further argument for the presence of this mechanisms early in evolution is that *in vitro* translation systems from all three domains of life have the ability to translate leaderless transcripts [20]. In contrast to all other known initiation mechanisms, initiation on leaderless transcripts requires the undissociated 70S/80S ribosome in addition to the initiator tRNA [21], [22].

A third mechanism is the so-called scanning mechanism. The small subunit of the ribosome binds to the 5′-end of the transcript with the help of translation initiation factors described below and subsequently linearly scans along the mRNA until the start codon is reached. Until now this mechanism has been exclusively found in eukaryotes.

A fourth initiation mechanism depends on so-called “Internal Ribosome Entry Sites” (IRES). IRES are folded structures embedded in 5′-UTRs that bind specific “IRES transacting factors” (ITAFs), which are involved in attracting the small subunit of the ribosome. IRES have been first observed in the 5′-UTRs of eukaryotic viruses. Subsequently it has been found that also cellular mRNAs of eukaryotes can contain IRES, which are much less studied. The fraction of IRES-containing transcripts varies widely, from about 3% to as much as 15%, partly because it is not straightforward to prove that an observed translation event was initiated on a *bona fide* IRES [5].

A fifth initiation mechanism operates on transcripts containing a 5′-UTR that is devoid of a SD sequence and an IRES and does not involve ribosomal scanning. It has been termed “novel mechanism” because the molecular details and factor dependence are still unknown [23] and will be called “SD-less mechanism” in this manuscript. Transcripts with 5′-UTRs lacking a SD sequence are found in archaea as well as in bacteria, e.g. about 1/3 of all transcripts are of this type both in *E. coli* and in *H. volcanii*.

It should be noted that these five initiation mechanisms (Table S1) are man-made categories and that several sorts of exceptions exist. For example, subcategories can be defined, mixed forms can exist on specific transcripts (e.g. IRES-dependent transcripts that make use of the 5′-cap), or two mechanisms can be used simultaneously and independently on a single transcript at least on artificial synthetic transcripts.

Moreover, the occurrence of translation initiation factors is very different in bacteria, archaea, and eukaryotes, indicating that the molecular mechanisms of translation initiation on the same types of mRNAs are not identical in the three phylogenetic lineages.

Bacteria contain just three translation initiation factors, IF1 – IF3. IF3 binds to the small ribosomal subunit. It is responsible for subunit dissociation and is an anti-association factor. IF2 binds the initiator tRNA and adjusts it in the ribosomal P site. This process is supported by IF1, which has a fidelity function and is bound to the ribosomal A site. IF2 as well as IF3 also have a fidelity function during this process [24]–[27].

In eukaryotes, many more initiation factors (eIFs) are involved in translation initiation than described for bacteria. For example, the 5′-cap of transcripts is recognized by aIF4F, a heterotrimer comprised of eIF4A, eIF4E and eIF4G. Several initiation factors, e.g. eIF2, eIF1 and eIF1A, and the initiator tRNA bind to the small subunit of the ribosome and thereby form the 43S initiation complex. This complex is recruited to the 5′-end of the messenger RNA because eIF4G interacts both with cap-binding factor eIF4E and the 43S complex. This complex then scans linearly along the transcript until the first AUG is reached. Then the large ribosomal subunit is recruited [28], [29]. Many natural and designed mutants, including human individuals with genetic diseases, underscore that linear scanning occurs [30].

Archaea possess more translation initiation factors than bacteria [7], [11]. The number is lower than in eukaryotes because most factors involved in forming the pre-initiation complex and binding to the cap structure like eIF3, eIF4E, eIF4F or eIF4G are lacking in accordance to the absence of a cap-structure at the 5′-end of transcripts. At least seven translation initiation factors, which contain up to three subunits, are described for archaea. They include orthologs to the bacterial/eukaryotic factors IF1/eIF1A and IF2/eIF5B, which are thus universally conserved in all three domains of life. But archaea also contain homologues to eukaryotic factors that are absent in bacteria, e.g. eIF1, eIF2, eIF4A, eIF5A, and eIF6.

The prediction of homology between archaeal and eukaryotic factors is based on sequence similarity. However, archaea and eukaryotes use a non-overlapping set of translation initiation mechanisms. Archaea use the SD-mechanism, the leaderless mechanism, and the SD-less mechanism, while higher eukaryotes use the scanning mechanism and the IRES-mechanism. Therefore, it is likely that some archaeal initiation factors (aIFs) and eukaryotic initiation factors (eIFs) might have different molecular functions despite their sequence similarity and their common origin. Analysis of archaeal translation initiation factors and comparison with their eukaryotic counterparts thus offers the potential to deepen the understanding of the evolution of translation initiation and discriminate between very early functions common to aIFs and eIFs and evolved functions developed during the evolution of the two lineages.

However, experimental studies on translation initiation in archaea are very limited, and almost all studies used *Sulfolobus solfataricus* as model organism [31]–[34]. *S. solfataricus* belongs to the kingdom of Crenarchaeota, and a systematic experimental analysis of translation initiation factors of a representative of the kingdom of Euryarchaeota would be desirable to complement these results. Therefore, this study concentrated on the investigation of translation initiation factors in haloarchaea using the model species *Haloferax volcanii*. In the genome sequence of *H. volcanii* 14 genes are annotated to code for putative translation initiation factors or subunits thereof. To gain a comprehensive insight, we attempted to delete all 14 genes coding for initiation factors. In cases where aIFs turned out to be essential for translation initiation, the corresponding gene products were conditionally depleted. The phenotypes of all deletion and depletion mutants were compared to that of the wild-type under various conditions. Furthermore, a translatome comparison of the wild-type and a deletion mutant was performed to investigate the effect of this mutation on gene expression. The results are discussed in comparison to the proposed functions of the initiation factors in *Sulfolobus* and in eukaryotes.

## Results

### Construction of deletion and depletion mutants

**Figure 1:**
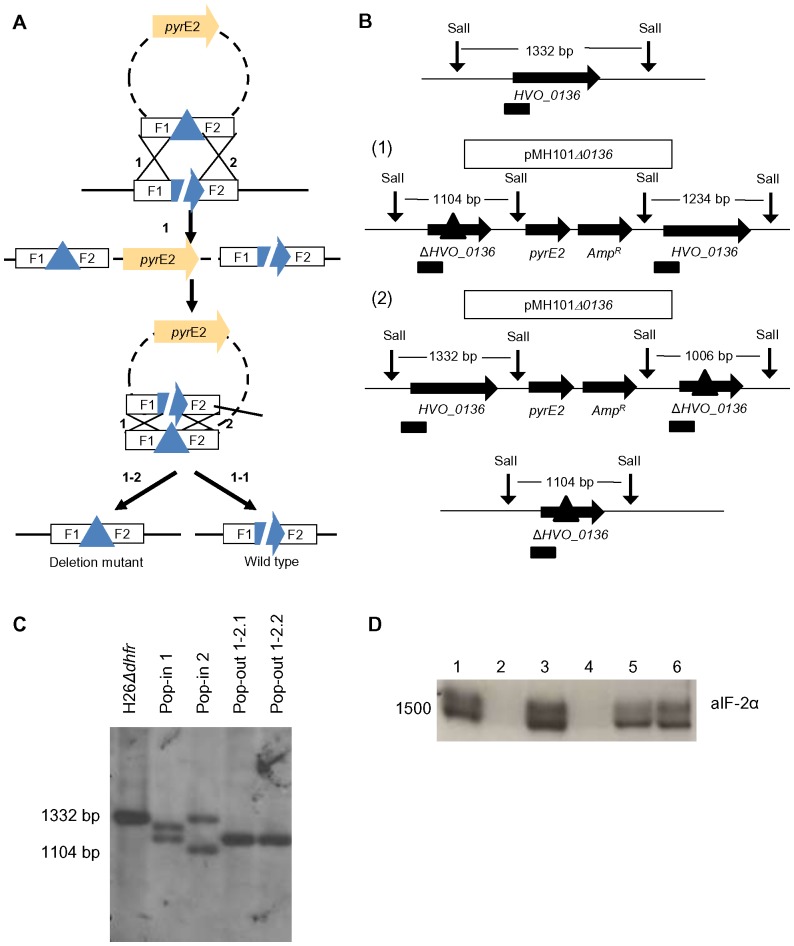
Generation and characterization of gene deletion mutants. A. Schematic overview of the Pop-In-Pop-Out method for mutant construction. The uracil auxotrophic strain H26 (*ΔpyrE2*) was transformed with the plasmid carrying the *pyrE2* gene and an *in frame* deletion version of the respective gene with upstream- and downstream sequences. Two possible homologous recombination events (1 and 2) can lead to integration of the plasmid into the genome (Pop-In), which can be selected by the absence of uracil. Pop-Out clones can be selected in the presence of uracil and 5-FOA, leading either to the wild-type (1-1) or to the deletion mutant (1–2). B. Genomic organizations of wild-type, Pop-In and Pop-Out mutants: one example. Schematic overviews of the genomic organizations around *HVO_0136* with the wild-type on top, the two possible Pop-In mutants in the middle and the Pop-Out mutant at the bottom. The integrated plasmid is shown above the genome of the Pop-In mutants. Relevant restriction sites are presented as vertical arrows and probes for Southern blot analysis are shown as boxes below the genome organization. C. Southern blot analysis of wild-type and mutants Verification of the wild-type, both Pop-In mutants and two positive deletion Pop-Out mutants of deletion mutant *HVO_0136* are shown from left to right. Wild-type and deletion fragments are indicated by their size. D. Verification of the absence of an *aIF2α* transcript in the deletion mutant The *aIF2α* and the two *aIF2β* deletion mutants were cultivated in complex media to mid-exponential growth phase. Cells were harvested, RNA was isolated and transcript levels were analyzed by Northern blot analysis using a probe complementary to the first 360 bp of the *aIF2α* gene. Samples: 1. H26*Δdhfr*, 2. H26*ΔdhfrΔ0699* (aIF2α), 3. H26*ΔdhfrΔ1678* (aIF2α), 4. H26*ΔdhfrΔ0699Δ1678* (aIF2α + aIF2β), 5. H26, 6. H26*Δ2242*.

In the genome sequence of *H. volcanii* 14 genes are annotated to code for translation initiation factors or subunits thereof (www.halolex.mpg.de). As a prerequisite for a comprehensive study of these factors the *dhfr* (dihydrofolate reductase) gene was deleted from the strain H26 to enable the application of the *dhfr* reporter gene in future experiments with the *aIF* gene deletion mutants. The *dhfr in frame* deletion mutant could be successfully constructed and was confirmed by analytical PCR and by Southern blot analysis (data not shown). Subsequently, it was attempted to generate single gene deletion mutants of all 14 *aIF* genes using the *Δdhfr* strain. The well-established Pop-In-Pop-Out method was used for all mutant constructions attempted in this study, which is schematically summarized in Fig. 1A [35], [36]. In total, 9 of the 14 genes could successfully be deleted, indicating that the respective genes were not essential at least under the standard growth conditions used for mutant construction. Mutant construction and its experimental verification are exemplified using the gene *HVO_0136* (*aIF1A-1*). Fig. 1B shows schematic overviews of the genomic organizations of the wild-type, the two possible Pop-In variants, and the deletion mutant after successful Pop-Out. The probe used for Southern blot analysis and the sizes of relevant restriction fragments used for Southern blot analysis are indicated. Fig. 1C shows the signals of a Southern blot analysis of the wild-type, two different Pop-In variants, and two Pop-Out clones derived from these variants. The genomic organization of the additional 8 *in frame* deletion mutants of *aIF* genes were also analyzed by Southern blot analyses (data not shown). Phenotypic characterization of all deletion mutants will be discussed below.

Also for the remaining 5 genes Pop-In mutants could be readily generated. However, the analysis of clones after Pop-Out selection revealed that all of them had wild-type genomes. In each case more than 100 clones were analyzed until it was concluded that these 5 genes were essential for *H. volcanii* (HVO numbers 0117, 1901, 1946, 1963, and 2300). The encoded gene products (aIF1, aIF2γ, aIF5A, aIF5B, aIF6) and their putative functions are described below.

It was especially unexpected that the gene for the α subunit of the heterotrimeric initiation factor aIF2 (*HVO_0699*) could be deleted, while the gene for the γ subunit (*HVO_1901*) turned out to be essential. It had been expected that all subunits of aIF2 would be essential, because this factor has been shown to be involved in binding the initiator tRNA in the archaeon *S. solfataricus*, which is also true for the orthologous factor eIF2 in eukaryotes. Therefore, in this case the successful construction of the deletion mutant was not only verified on the DNA level, but also on the RNA level. Fig. 1D shows a Northern blot analysis that underscores that the transcript of *HVO_0699* is readily detectable in the wild-type and a deletion mutant of the gene for the β subunit, but undetectable in mutants lacking the gene *HVO_0699*.

**Figure 2:**
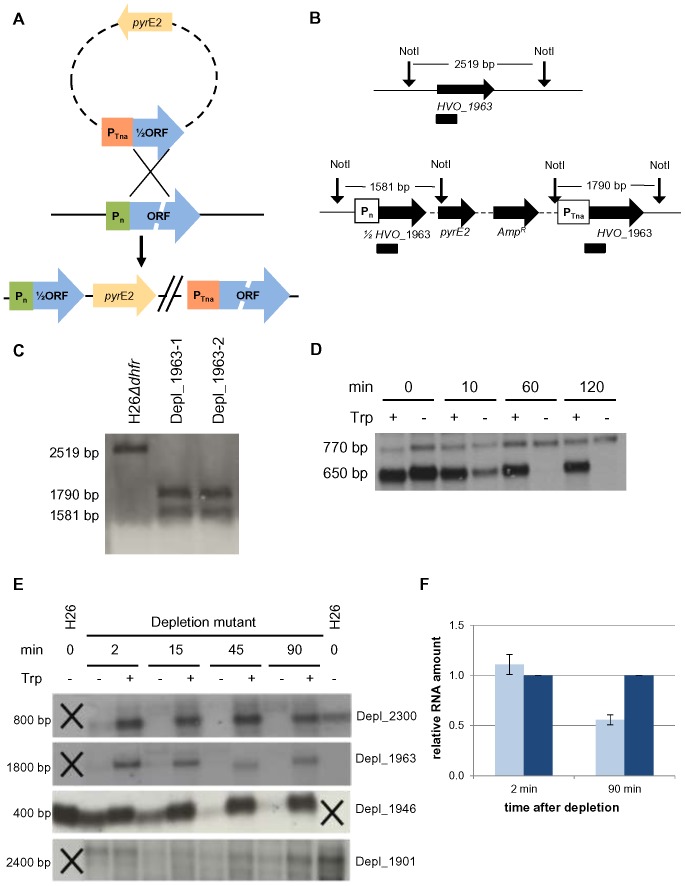
Construction and characterization of the conditional depletion mutants. A. Schematic overview of the strategy to construct conditional depletion mutants. Plasmids containing the *pyrE2* gene and half of the gene of interest behind a tryptophan inducible promoter were used to transform *H. volcanii* H26*Δdhfr*. Homologous recombination between the plasmid-encoded gene and the native gene on the chromosome can be selected in the absence of uracil. As a result, a full-length functional copy is placed under the control of the tryptophan-inducible promoter, while the native promoter drives expression of the truncated, non-functional version. B. Schematic overview of the genomic organizations of the wild-type and one depletion mutant. Schematic overview of the genomic organization around *HVO_1963* in the wild-type (on top) and the conditional depletion mutant (at the bottom). Relevant restriction sites are presented as vertical arrows and probes for Southern blot analysis are shown as boxes below the genome organization. C. Southern blot analysis of the wild-type and depletion mutants. Southern blot analysis of the wild-type and two depletion mutants (from left to right). Sizes of hybridizing fragments are indicated to the left. D. Analysis of conditional expression of *HVO_0117*. Depletion mutant of *HVO_0117* was grown in synthetic medium on glucose with 100 µg/ml tryptophan. For depletion, cells were washed in 2.1 M NaCl and inoculated in medium with or without tryptophan. RNA was isolated directly after depletion and 10 min, 60 min and 120 min later. Transcript levels were analyzed by Northern blot analysis and transcript sizes are indicated on the left. E. Analysis of conditional expression of four additional depletion mutants. Depletion mutants were grown to mid-exponential phase in synthetic medium with CAS and 100 µg/ml tryptophan. After washing, cells were inoculated in medium with or without tryptophan. RNA was isolated 2 min, 15 min, 45 min and 90 min after depletion. (A) Transcript levels were analyzed by Northern blot analyses with probes specific for the respective genes. Black crosses indicate that there is no wild-type in this column. Transcript sizes and the HVO number of the depletion mutant are shown on the left and on the right. F. Quantitative RT-PCR analysis of transcript depletion in mutant *HVO_1901* Real-time analysis of depletion mutant Depl_1901 2 min and 90 min after depletion. The mutant cultivated with tryptophan is shown in dark blue and normalized to 1, the color code for the depleted mutant without tryptophan is defined with light blue (n = 3).

Essential genes cannot be deleted, but the functions of their gene products can be studied *in vivo* when conditional depletion is possible. Therefore, conditional depletion mutants of all five essential genes were generated. The experimental design is shown schematically in Fig. 2A. The 5′-halves of the genes were cloned downstream of the promoter of the *tna* gene into a suicide vector for haloarchaea. Transformation of a suitable *H. volcanii* strain and selection for the presence of the *pyrE2* gene results in mutants that have integrated the vector into their genome via homologous recombination. Their genome contains a non-functional shortened copy of the gene under the control of the native promoter and a functional copy of the respective *aIF* gene under the control of the *tna* promoter. As a consequence, the expression of the functional copy can be regulated by the presence or absence of tryptophan. The analysis of the successful construction of the mutants is exemplified for gene *HVO_1963*. Fig. 2B schematically shows the genomic organization of the wild-type and the depletion mutant, and Fig. 2C shows the result of a Southern blot analysis. The successful generation of the other four depletion mutants was also verified by Southern blot analyses (data not shown). It has been shown that the activity of the *tna* promoter in the absence of tryptophan is extremely low when it is integrated into the genome [37]. However, the transcript level is not only determined by the rate of transcription, but also by the rate of degradation. Therefore, the velocity of depletion after the removal of tryptophan might vary and thus it was determined for all five genes. As a first example, the results for gene *HVO_0117* (aIF6) are shown in Fig. 2D. The transcript under the control of the *tna* promoter has a size of 650 nt. Tryptophan removal led to an about threefold reduction of the transcript level within 10 minutes, and after 60 minutes it was undetectable by Northern blot analysis. In this case the probe detected a second transcript with a size of 770 nt. This transcript is due to the localization of the gene *HVO_0117* in an operon where the native promoter now drives the transcription of a polycistronic transcript containing *HVO_0115*, *HVO_0116* and the deletion version of *HVO_0117*. The level of this transcript is not influenced by the presence or absence of tryptophan, but of course it does not encode a functional aIF6 (compare Fig. 2A).

The results of the Northern blot analyses for the remaining four genes are shown in Fig. 2E, and it is obvious that the half lives of the four transcripts are very different. The transcript of *HVO_2300* (aIF5A) is severely decreased already after 2 minutes of tryptophan removal and it is undetectable 15 minutes after depletion. Similarly, the transcript level of *HVO_1963* (*aIF5B*) is severely reduced already after 2 minutes of depletion, but a very low concentration is still visible after 15 minutes of depletion. In contrast, the transcript of *HVO_1946* (*aIF1*) is much more stable and a small transcript level is still visible after 90 minutes of depletion. However, by far the highest transcript stability was observed for transcript *HVO_1901* (*aIF2γ*), which was not severely reduced even after 90 minutes of depletion. To investigate this further, transcript levels of *HVO_1901* were quantified using qRT-PCR (Fig. 2F). The results underscored the high transcript stability revealed by Northern blot analysis, about 50% of the transcript remained after 90 minutes of tryptophan removal. Nevertheless, the results show that transcript depletion was successful in all five cases, albeit the velocity was remarkably different.

Four genes that could be successfully deleted were in fact two pairs of orthologous genes coding for aIF1A and aIF2β, respectively. It seemed possible that these two factors are redundantly encoded in *H. volcanii* and the construction of single gene deletion mutants is possible in spite of the essential function of these aIFs. Therefore, it was attempted to generate double deletion mutants of genes *HVO_0136/HVO_A0637* (*aIF1A*) and *HVO_1678/HVO2242* (*aIF2β*). In the former case, 138 Pop-Out clones were analyzed, and in the latter case 186 Pop-Out clones were analyzed, all of which turned out to be single deletion mutants. Based on these results we concluded that also aIF1A and aIF2β are essential translation initiation factors for *H. volcanii* and that the successful generation of single deletion mutants was only possible because these two factors are encoded by two orthologous genes that can replace one another at least under the standard conditions used for mutant construction. This interpretation was corroborated by a control experiment. The attempt to delete the gene *HVO_1678* (*aIF2β*) in the background of the single deletion mutant of *HVO_0699* (*aIF2α*) was readily successful, showing that a mutant strain lacking a gene for aIF2α and containing only one gene for *aIF2β* and one gene for *aIF2γ* is viable.

In addition, it was attempted to construct a double deletion mutant of the genes HVO_1934 and *HVO_2706*, which encode proteins with similarity to the α and the δ subunits of the eukaryotic initiation factor *aIF2B*. The double mutant could readily be constructed. As will be discussed below, these results shade doubt on the annotation that these genes encode *bona fide* translation initiation factors.

The phenotypes of the deletion and depletion mutants have been analyzed and compared to that of the wild-type under various conditions, and the results are presented in the following paragraphs separately for the dispensable genes, the redundantly encoded and essential genes, and the bona fide essential genes.

### Phenotypic characterization of single gene deletion mutants

Recently a method to cultivate *H. volcanii* in microtiter plates was established [38], which enabled the comparison of growth of the wild-type and the nine deletion mutants in triplicates simultaneously under a variety of different conditions. Six different C-sources were analyzed, i.e. complex medium containing yeast extract and tryptone and synthetic medium with casamino acids, glucose, pyruvate, sucrose and acetate, respectively, as sole carbon and energy sources. In addition, cells were grown at three different temperatures both in complex medium and in synthetic medium with glucose as carbon source. Cultures were grown at the optimal temperature of 42°C, at the reduced temperature of 30°C, and near the upper temperature limit of growth at 50°C. Furthermore, cultures were grown at three different salt concentrations, the optimal concentration of 2.1 M NaCl, at 4.0 M NaCl, and at 0.7 M NaCl, which represents the lowest salt concentration *H. volcanii* can tolerate. All growth curves are shown in Supplementary Figures S1 and S2, and selected growth phenotypes are discussed below for the dispensable initiation factors and the redundantly encoded but essential initiation factors (see Table S2 for detailed information).

### Dispensable initiation factors

**Figure 3:**
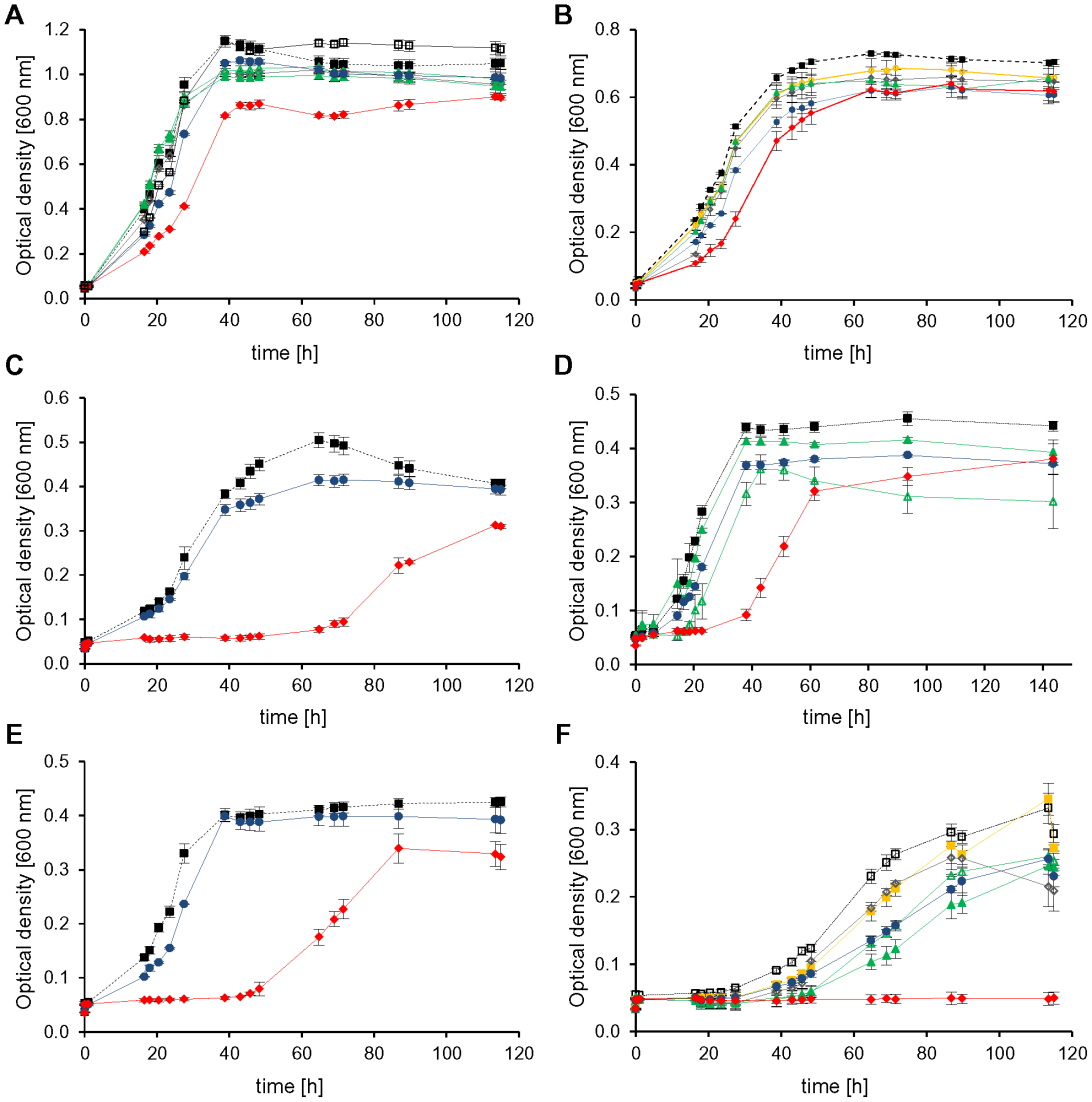
Phenotypical characterization of *aIF* deletion mutants in media with different carbon sources. Nine gene deletion mutants and the *H26Δdhfr* wild-type were cultivated in microtiter plates on six different C-sources. For clarity only growth curves of the wild-type (filled black squares, dotted line) and those mutants with a phenotypic variation from the wild-type are shown. Cultures were grown in complex medium (A) and synthetic medium with CAS (B), glucose (C) pyruvate (D), sucrose (E) and acetate (F) as carbon source. Average results from triplicate cultures and their standard deviations are shown. The color code is defined with dotted lines for the wild-type and solid lines for the mutants: Wild-type (black squares), Δ0699 (aIF2α, filled red diamonds), Δ1333 (eIF4A homolog, open grey diamonds), Δ1934 (aIF2Bα, open blue squares), Δ2706 (aIF2Bδ, open yellow circles), ΔB0284 (aIF1–2, open black squares), Δ0136 (aIF1A-1, filled blue circles), ΔA0637 (aIF1A-2, filled yellow squares), Δ1678 (aIF2β-1, open green triangles), Δ2242 (aIF2β-2, filled green triangles).

**Figure 4:**
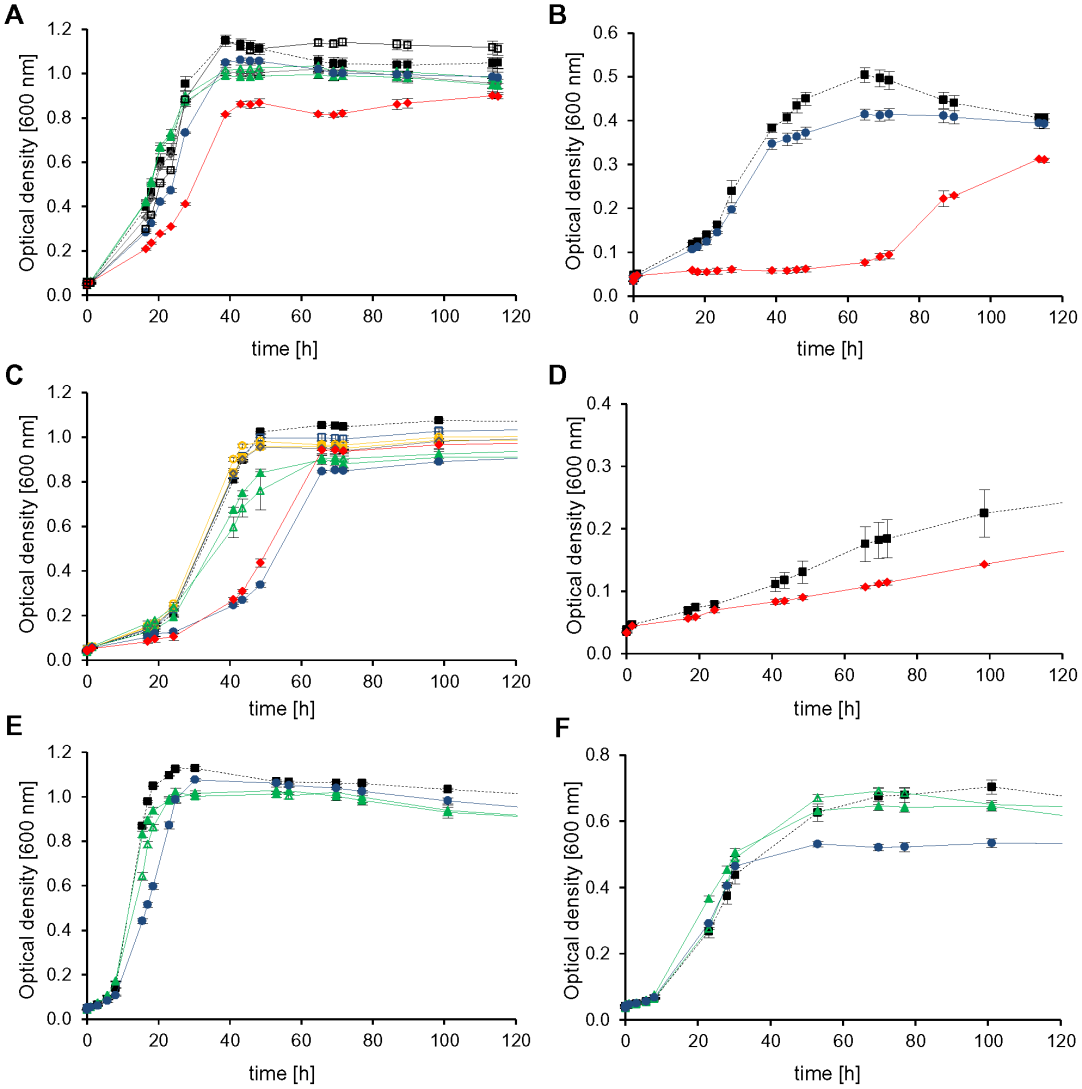
Growth of *aIF* deletion mutants at different temperatures. Cultures were grown at different temperatures either in complex medium (A, C, E) or in synthetic medium with glucose (B, D, F). They were grown at the standard temperature of 42°C (A, B), at the reduced temperature of 30°C (C, D) and the elevated temperature of 50°C (E, F). Average results from triplicate cultures and their standard deviations are shown. The color code for the mutants is given in Figure 3.

**Figure 5:**
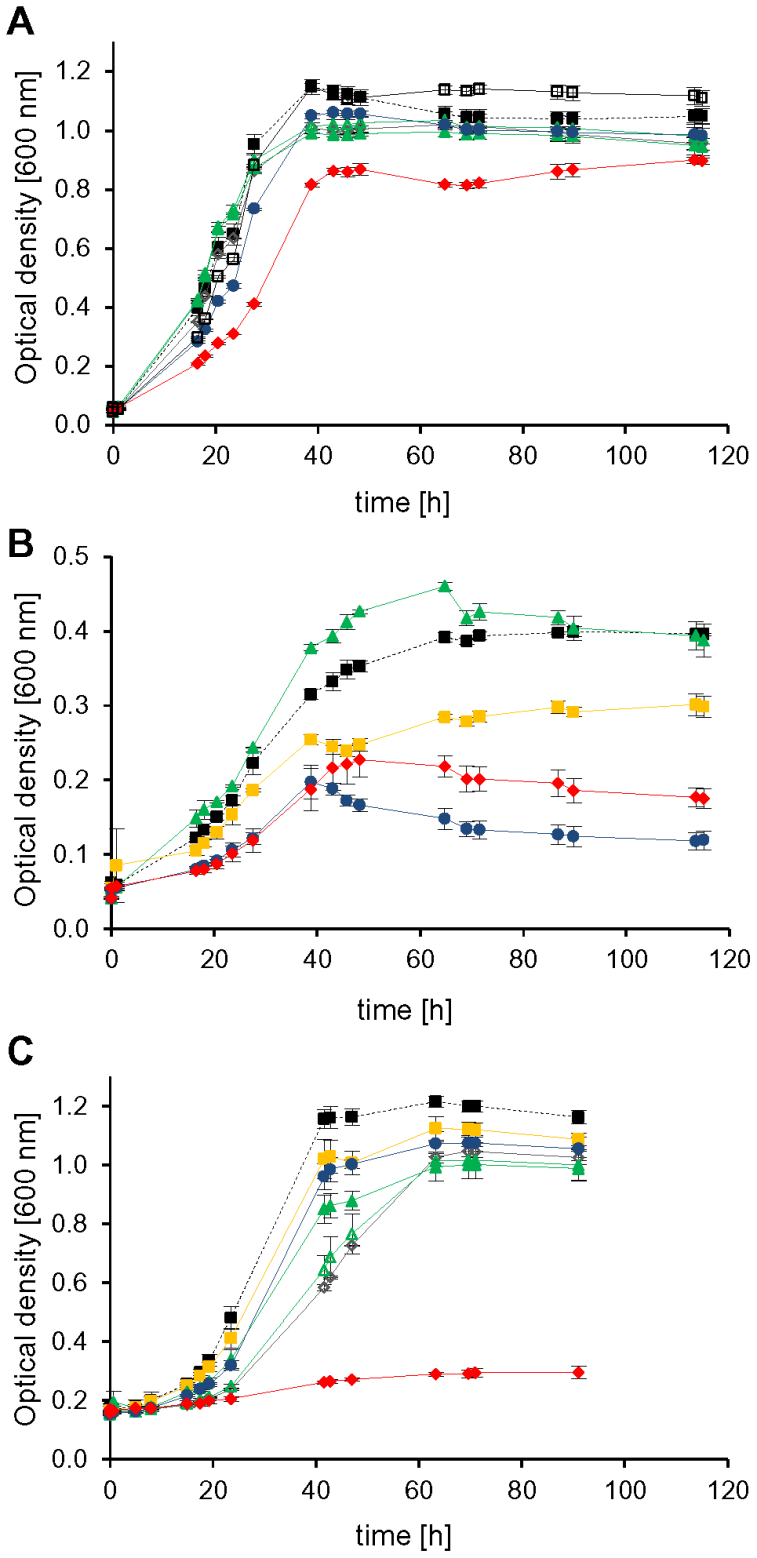
Growth of *aIF* deletion mutants at different salt concentrations. Cultures were grown at the optimal salt concentration of 2.1(A), the reduced salt concentration of 0.7 M NaCl (B), and the elevated salt concentration 4 M NaCl (C). Average results from triplicate cultures and their standard deviations are shown. The color code for the mutants is given in Figure 3.

Five non-redundant genes could be successfully deleted indicating that they do not encode essential proteins. However, the phenotypes of the deletion mutants differed remarkably. The deletion mutant of *HVO_0699*, encoding aIF2α had a very pleiotrophic phenotype and differed from the wild-type under nearly all conditions tested. The mutant showed a mild phenotype during growth in complex medium and casamino acids, a severe phenotype during growth on glucose, sucrose and pyruvate, and it could not grow at all on acetate (Fig. 3, red curves). The mutant lacking aIF2α was not only compromised at the optimal temperature of 42°C, but grew also worse than the wild-type at the reduced temperature of 30°C (Fig. 4, red curves). In contrast, it grew indistinguishable to the wild-type at 50°C (Fig. S2 C+D). In complex medium the growth defect of the mutant lacking aIF2α was only moderate at the optimal salt concentration of 2.1 M NaCl, but very severe at 0.7 M NaCl and the mutant was completely unable to grow at 4.0 M NaCl (Fig. 5, red curves). In summary, the phenotypic analysis revealed that aIF2α is not essential but very important for *H. volcanii* under many conditions and its importance is correlated to the environmental conditions, e.g. aIF2α is essential for growth on acetate.

In contrast to the severe effects of deletion of the gene *HVO_0699* encoding aIF2α deletion of the remaining four non-essential genes did not result in any or only very small phenotypic differences to the wild-type. The deletion of *HVO_B0284* encoding aIF1 resulted in very small differences to the wild-type only during growth in complex medium and in synthetic medium on acetate (Fig. 3, solid line, open black squares). However, it should be noted that *H. volcanii* contains another gene encoding aIF1 (*HVO_1946*), which turned out to be essential. Therefore, it might well be that the annotation of *HVO_B0284* is not correct and it encodes a protein not involved in translation initiation with a different, non-essential function.

**Figure 6:**
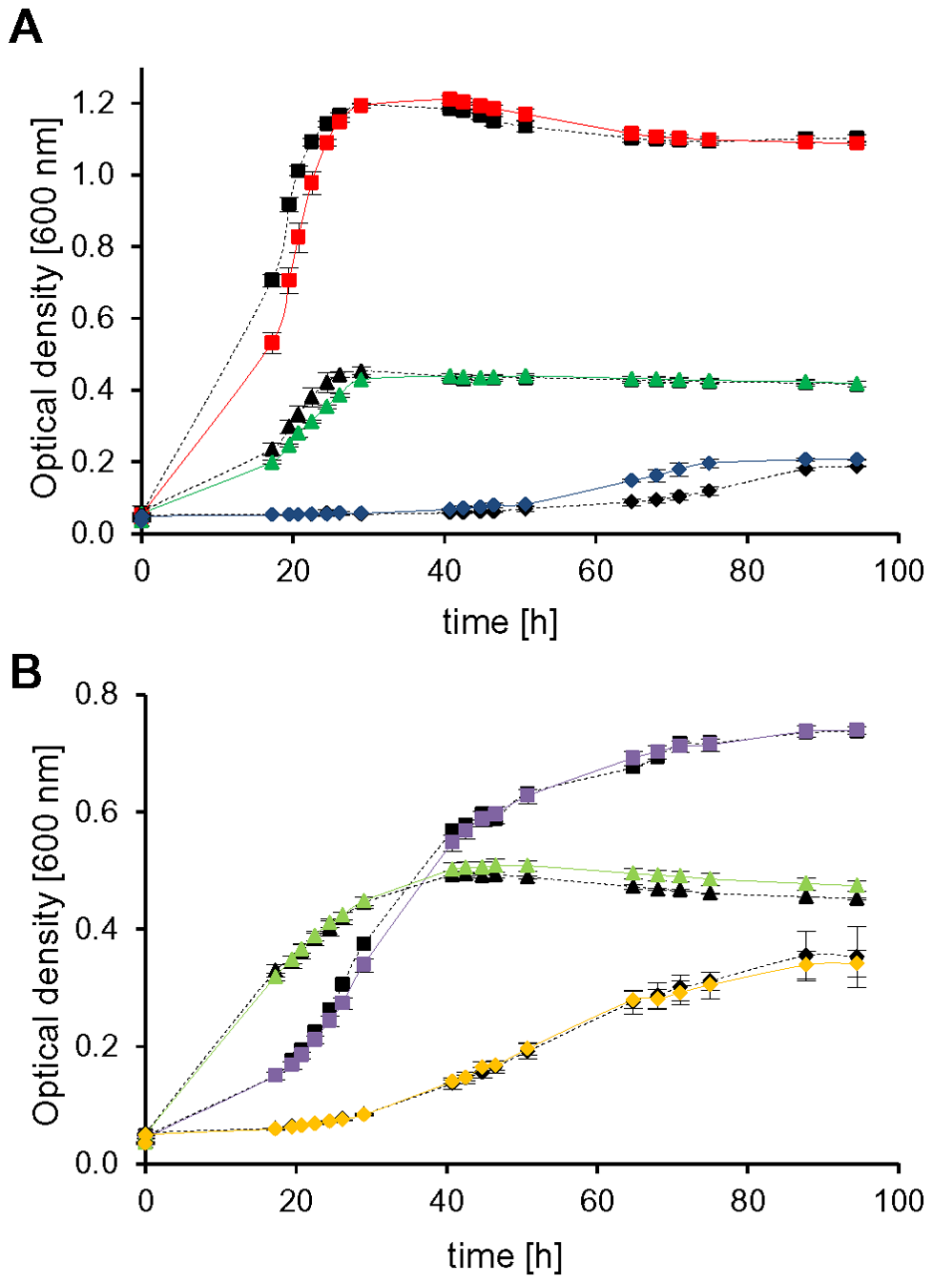
Phenotypical characterization of an *aIF2B* double deletion mutant. The *aIF2B* double deletion mutant (*HVO_1934, HVO_2706*) was grown in medium with six different carbon sources and growth was compared to that of the wild-type (dotted black lines). Growth conditions with phenotypic differences between double mutant and wild-type are shown in (A), conditions under which double mutant and wild-type grew indistinguishably are shown in (B). Average results from triplicate cultures and their standard deviations are shown. The color code is defined with red squares for complex media, green triangles for sucrose media, and blue diamonds for xylose in Figure A. In B glucose (violet squares), CAS (light green triangles) and acetate (yellow diamonds) are shown.

The genes *HVO_1934* and *HVO_2706* encode proteins with similarities to the α and δ subunits of the eukaryotic initiation factor eIF2B. The single deletion mutants did not show phenotypic differences to the wild-type under any of the 12 different conditions tested. Therefore, a double deletion mutant of *HVO_1934* and *HVO_2706* was constructed. However, also the double deletion mutant grew indistinguishable from the wild-type under various conditions (Fig. 6), indicating that the two proteins are not involved in or not important for translation initiation.

The gene *HVO_1333* encodes a protein with similarity to the eukaryotic initiation factor eIF4A. The deletion mutant grew indistinguishable from the wild-type under nearly all conditions, only small differences could be observed during growth on casamino acids as carbon source and in complex medium with 4 M NaCl (Table 1). It can be concluded that also the protein encoded by *HVO_1333* is either not involved or not important for translation initiation in *H. volcanii*.

**Table 1:** Dispensable and redundantly encoded genes for translation initiation factors of *H. volcanii* and the phenotypes of single gene deletion mutants.

	Condition	Lag phase	t_d_ [h]	t_d_ [% wt]	g.y. [% wt] (s.d. [%])
**DISPENSABLE**					
**H26** ***ΔdhfrΔ0699*** ** (aIF2α)**					
	2.1 M complex	equal	**11.3**	**194%**	**86% (1%)**
	CAS	**elongated**	**10.4**	**121%**	88% (5%)
	glucose	**elongated**	**22.7**	**176%**	**76% (2%)**
	pyruvate	**elongated**	**12.8**	**120%**	94% (5%)
	sucrose	**elongated**	**15.3**	**151%**	94% (5%)
	acetate	n.a.	**n.a.**	**n.a.**	**17% (18%)**
	0.7 M NaCl	**elongated**	**17.1**	**135%**	**44% (8%)**
	4 M NaCl	**elongated**	**52.5**	**429%**	**25% (7%)**
	30°C complex	**elongated**	**13.9**	**153%**	**92% (1%)**
	30°C glucose	**elongated**	**66.9**	**198%**	**72% (2%)**
**H26** ***ΔdhfrΔ1333*** ** (eIF4A homolog)**					
	CAS	**elongated**	6.3	74%	92% (3%)
	4 M NaCl	**elongated**	14.0	115%	**88% (2%)**
**REDUNDANTLY ENCODED BUT ESSENTIAL**					
**H26** ***ΔdhfrΔ0136*** ** (aIF1A-1)**					
	CAS	equal	**9.4**	**110%**	86% (4%)
	acetate	equal	**26.8**	**132%**	**79% (7%)**
	0.7 M NaCl	**elongated**	**17.0**	**134%**	**30% (11%)**
	4 M NaCl	equal	12.4	101%	**91% (4%)**
	30°C complex	**elongated**	**15.1**	**165%**	**86% (0%)**
	50°C complex	**elongated**	**4.0**	**135%**	94% (2%)
	50°C glucose	equal	7.2	95%	**80% (1%)**
**H26** ***ΔdhfrΔA0637*** ** (aIF1A-2)**					
	4M NaCl	equal	13.1	107%	**94% (2%)**
**H26** ***ΔdhfrΔ1678*** ** (aIF2β-1)**					
	CAS	**elongated**	5.8	68%	95% (6%)
	acetate	**elongated**	19.6	96%	86% (4%)
	4 M NaCl	**elongated**	13.9	113%	**86% (5%)**
	30°C complex	equal	**12.0**	**132%**	**89% (2%)**
	50°C complex	equal	**4.0**	**133%**	90% (3%)
	30°C glucose	**elongated**	34.9	103%	102% (14%)
**H26** ***ΔdhfrΔ2242*** ** (aIF2β-2)**					
	acetate	**elongated**	23.4	115%	83% (5%)
	4 M NaCl	equal	13.4	109%	**85% (4%)**
	30°C complex	equal	9.9	109%	**86% (1%)**
	50°C glucose	equal	**6.6**	**87%**	91% (2%)

td = doubling time; g.y. = growth yield; s.d. = standard deviation; n.a. = not available (no growth of the mutant); bold print = phenotypes; equal = equal to the wild-type.

### Redundantly encoded but essential initiation factors

As described above the two factors aIF1A and aIF2β are each encoded by two orthologous genes (*HVO_0136/HVO_A0637* and *HVO_1678/HVO_2242*). In both cases only single gene deletion mutants could be generated, but no double mutants, indicating that aIF1A and aIF2β are essential. However, in both cases the single deletion mutants exhibited phenotypic differences to the wild-type at least under specific conditions, therefore it seems that in both cases the two orthologs have overlapping yet not identical functions. For example, the single deletion mutants of both genes encoding aIF1A showed a rather severe phenotype at the low salt concentration of 0.7 M NaCl (Fig. 5B, *HVO_0136* – filled blue circles, *HVO_A0637* – filled yellow squares). During growth on acetate as sole carbon source both single deletion mutants exhibited a milder but clearly detectable phenotype (Fig. 3F, blue circles and yellow squares). Notably, only one of the two single *aIF1A* deletion mutants had a rather severe growth defect in complex medium at 30°C (blue circles), while the other mutant (yellow squares) grew nearly indistinguishable from the wild-type. Under all remaining conditions both single *aIF1A* deletion mutants grew very similar or indistinguishable from the wild-type. All phenotypic differences are summarized in Table 1.

Similarly, the single *aIF2β* deletion mutants exhibited clear phenotypic differences to the wild-type only under a few of the tested conditions. Both mutants had a clear growth defect during growth on acetate as sole carbon source (Fig. 3F, *HVO_1678* – open green triangles, *HVO_2242* – filled green triangles). In addition, both had a lower growth yield at 4.0 M NaCl (Fig. 5C, open and filled green triangles). Under all other conditions, both single *aIF2β* deletion mutants grew very similar or identical to the wild-type (Fig. 3–5). All phenotypic differences are summarized in Table 1.

The results showed that the two aIF2β orthologs could replace one another for translation initiation at least at all essential transcripts, but the phenotypes of the two single mutants indicated that the two aIF2βs act at an overlapping but not identical spectrum of transcripts. To test this idea, it was analyzed whether the absence of *HVO_2242* has any effect on the translatome of *H. volcanii*. Cultures of the mutant Δ*HVO_2242* and the wild-type were grown in complex medium to mid-exponential growth phase. Cytoplasmic extracts were generated and sucrose density gradients were used to separate free, non-translated transcripts and polysome-bound transcripts. RNA was isolated from both fractions and compared using a DNA microarray. The results were compared for the Δ*HVO_2242* mutant and the wild-type and all genes with an at least twofold deviation of translational efficiency in the mutant compared to the wild-type are listed in Table 2. Values of <0.5 represent transcripts that are less efficiently translated in the mutant, values of >2 represent transcripts that are better translated in the mutant. The highest difference was found for the second gene for aIF2β (*HVO_1678*), which was tenfold up-regulated in the absence of *HVO_2242*. This underscored that the two orthologs are not independent from one another, fulfill overlapping functions and can replace on another. Only very few additional genes were differentially translated in the wild-type and the deletion mutant. Therefore, in contrast to the genes for the other two subunits, aIF2α and aIF2γ, aIF2β seems to be redundantly encoded in the genome of *H. volcanii*. However, there was one example of a distinctive difference between the two strains. About half of the spots with an at least twofold reduced translation efficiency in the *HVO_2242* deletion mutant represent genes that belong to the ATP-synthase operon (*HVO_0311 – HVO_0317*). Therefore, it was analyzed whether this resulted in a reduced ATP level in the mutant compared with the wild-type. However, at least in mid-exponential growth phase in complex medium (the condition of the translatome comparison) no difference in the ATP level of mutant and wild-type could be detected. It remains to be clarified whether the translatome differences between mutant and wild-type are greater under conditions when the single mutants exhibit a phenotype, e.g. during growth on acetate.

**Table 2:** *H. volcanii* genes with differential translational efficiencies between the *HVO_2242* deletion mutant and the wild-type*.

Identifier	Gene name	Coding region	Translational efficiency mutant/wt		
			mean	s.d.	n
**HVO_1678**	*tif2b*	*HVO_1678*	**9.97**	0,02	6
**HVO_A0047**	*uspA26*	*HVO_A0047*	**3.02**	0,07	3
**sRNA22anti**			**2.84**	0,05	4
**sRNA22anti**			**2.79**	0,05	3
**432-B05**	*mhpD*	*HVO_2244*	**2.52**	0,08	6
**sRNA45**			**2.47**	0,12	6
**sRNA45**			**2.35**	0,13	6
**sRNA45**			**2.28**	0,11	6
**sRNA45**			**2.27**	0,11	4
**437-F11**	*citB1, acnA*	*HVO_0541*	**2.18**	0,12	3
**441-D11**	*capB*	*HVO_B0153*	**2.16**	0,14	4
**433-G05**	*top6A*	*HVO_1570*	**0.48**	0,50	6
**439-A06**	*atpF, atpA*	*HVO_0315-0316*	**0.46**	0,31	6
**435-D08**	*rpoB1*	*HVO_0348*	**0.46**	0,58	6
**432-B01**		*HVO_1290*	**0.46**	0,61	6
**434-E09**	*atpA, atpB*	*HVO_0316-0317*	**0.46**	0,45	6
**433-A11**	*atpC, atpF*	*HVO_0314-0315*	**0.45**	0,44	6
**448-F01**	*atpA, atpB*	*HVO_0316-0317*	**0.45**	0,51	6
**433-B07**	*atpC, atpF*	*HVO_0314-0315*	**0.44**	0,38	6
**sRNA57sense**			**0.43**	0,31	6
**448-G05**	*atpF, atpA*	*HVO_0315-0316*	**0.43**	0,50	6
**459-C10**	*atpI, atpK, atpE*	*HVO_0311-0313*	**0.43**	0,65	6
**sRNA450**			**0.41**	0,72	5
**sRNA57sense**			**0.40**	0,63	6
**sRNA529**			**0.39**	0,68	5
**460-C11**	*pstS2, pstC2*	*HVO_A0447-0448*	**0.33**	0,66	6

* All genes are tabulated which have a translation efficiency of mutant/wt of ≥2, if they have a more than twofold higher efficiency in the mutant, or ≤0.5, if they have a more than twofold lower efficiency in the mutant. The translational efficiency is calculated from the quotient of a transcript in the polysomal fraction and in the fraction of free, untranslated RNA. ORF numbers and gene names were taken from Halolex (halolex.mpg.de).

s.d. = standard deviation, n = number of experiments.

### Essential initiation factors

The five essential genes are coding for aIF1, aIF2γ, aIF5A, aIF5B, and aIF6, indicating that these initiation factors are especially important in haloarchaea. They were further analyzed using conditional depletion mutants. Northern blot analysis revealed that the transcripts of four genes could successfully be depleted in the absence of tryptophan to an undetectable level within 90 minutes or faster. While the protein levels could not be directly quantified, it can be expected that transcript depletion results in protein dilution in successive generations even if the proteins would be indefinitely stable. The only exception was the transcript of *HVO_1901* that could only partially be depleted after 90 minutes, which was shown by Northern blot analysis and qRT-PCR. Therefore, the results for the depletion of *HVO_1901* encoding aIF2γ should be regarded with care.

To characterize the effects of the depletion of the five essential translation initiation factors, pre-cultures were grown in the presence of tryptophan to mid-exponential growth phase and used to inoculate cultures that were grown in the absence and presence of tryptophan, respectively. Growth in synthetic media with five different carbon sources and at high salt conditions was tested and all growth curves are shown in Supplementary Figures S3 (see Table 3 and Table S3 for detailed information).

**Table 3:** Essential translation initiation factors of *H. volcanii* and the phenotypes of conditional depletion mutants with (d) or without (nd) depletion.

	d/nd	Condition	Lag phase	t_d_ [h]	t_d_ [% of wt]	t_d_ [% of nd]	g.y. [% wt] (s.d. [%])	g.y. [% nd]
**ESSENTIAL**								
**H26** ***Δdhfr*** **-depl_1946 (aIF1)**								
	d	glucose	equal	**31.6**	**319%**	**365%**	**68% (10%)**	**83%**
	d	pyruvate	equal	**22.5**	**320%**	**418%**	**80% (5%)**	**70%**
	d	sucrose	equal	**14.4**	**223%** *	**276%** *	**69% (1%)**	**73%**
	d	glycerol	equal	**23.3**	**503%**	**429%**	59% (7%)	78%
	d	CAS	equal	**14.0**	**180%** *	**170%** *	**53% (13%)**	**63%**
	d	4 M NaCl CAS	equal	**21.0**	**201%**	**149%**	96% (1%)	**87%**
	nd	glucose	equal	**8.6**	**90%**	-	**84% (4%)**	-
**H26** ***Δdhfr*** **-depl_1901 (aIF2γ)**								
	d	glucose	equal	9.1	92%	**72%**	117% (2%)	**130%**
	d	CAS	equal	6.9	101%	**96%**	124% (1%)	101%
	nd	glucose	equal	**12.6**	**131%**	-	**92% (2%)**	-
**H26** ***Δdhfr*** **-depl_2300 (aIF5A)**								
	d	glucose	equal	9.8	99%	116%	**67% (3%)**	**94%**
	d	pyruvate	equal	7.0	99%	**138%**	105% (3%)	**88%**
**H26** ***Δdhfr*** **-depl_0117 (aIF6)**								
	d	glycerol	**elongated**	**8.5**	**183%**	**132%**	46% (10%)	60%
	nd	glycerol	**elongated**	6.4	126%	-	84% (6%)	-

d = depleted; nd = non-depleted; t_d_ = doubling time; g.y. = growth yield; s.d. = standard deviation, bold print = phenotypes; equal = equal to the wild-type;

* = these values are not related to the wild-type shown in this table but to a separate experiment.

**Figure 7:**
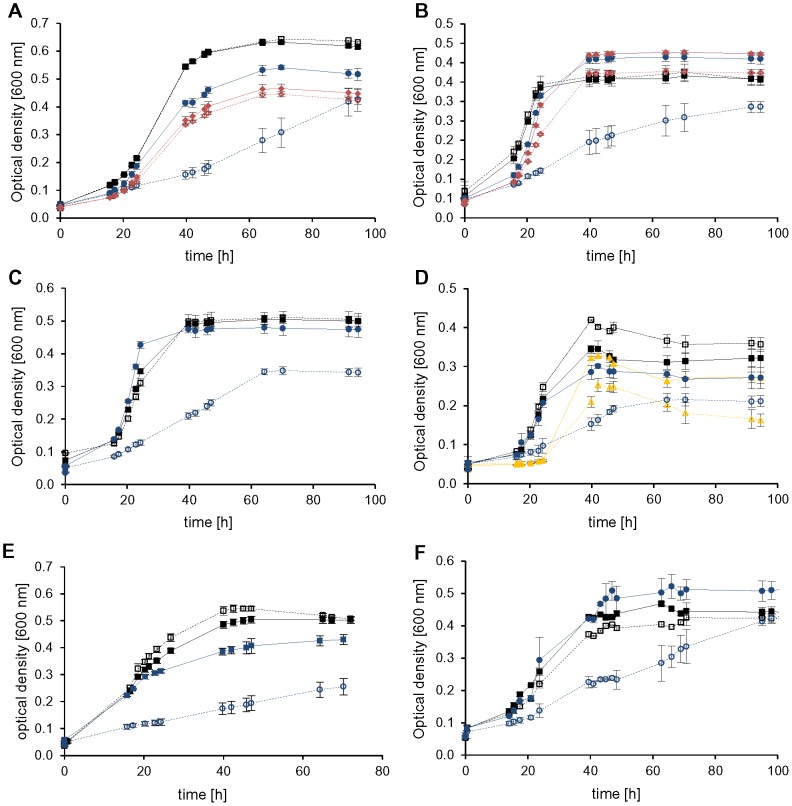
Phenotypical characterization of conditional depletion mutants. Cells were cultivated in synthetic medium with the optimal NaCl concentration of 2.1(A), pyruvate (B), sucrose (C), glycerol (D), and casamino acids (E) as carbon source, respectively. (F) shows growth in synthetic medium with casamino acids at the elevated salt concentration of 4 M NaCl. Average results from triplicate cultures and their standard deviations are shown. The color code is defined with dotted lines and open symbols for the wild-type (black squares) and depleted mutants cultivated without tryptophan, with solid lines and filled symbols for the wild-type and mutants cultivated with tryptophan. The following colors are used for the mutants: Depl_0117 (aIF6, yellow triangles), Depl_1946 (aIF1, blue circles), Depl_2300 (aIF5A, red diamonds).

As expected, mutant *HVO_1946* grew much worse in the absence than in the presence of tryptophan under all six conditions, underscoring the important role of aIF1 for *H. volcanii* (Fig. 7, dotted blue line versus solid blue line). However, even in the absence of tryptophan residual growth was observed (possible reasons are discussed below). In the presence of tryptophan the mutant grew worse than the wild-type under some conditions and better than the wild-type under other conditions (Fig. 7, solid blue versus solid black line). Most probable the native promoter of *HVO_1946* in the wild-type is differentially regulated under various conditions, so that the protein level of *HVO_1946* in the mutant, which is under the control of the tna promoter, is sometimes higher and sometimes lower than that of the wild-type.

**Figure 8:**
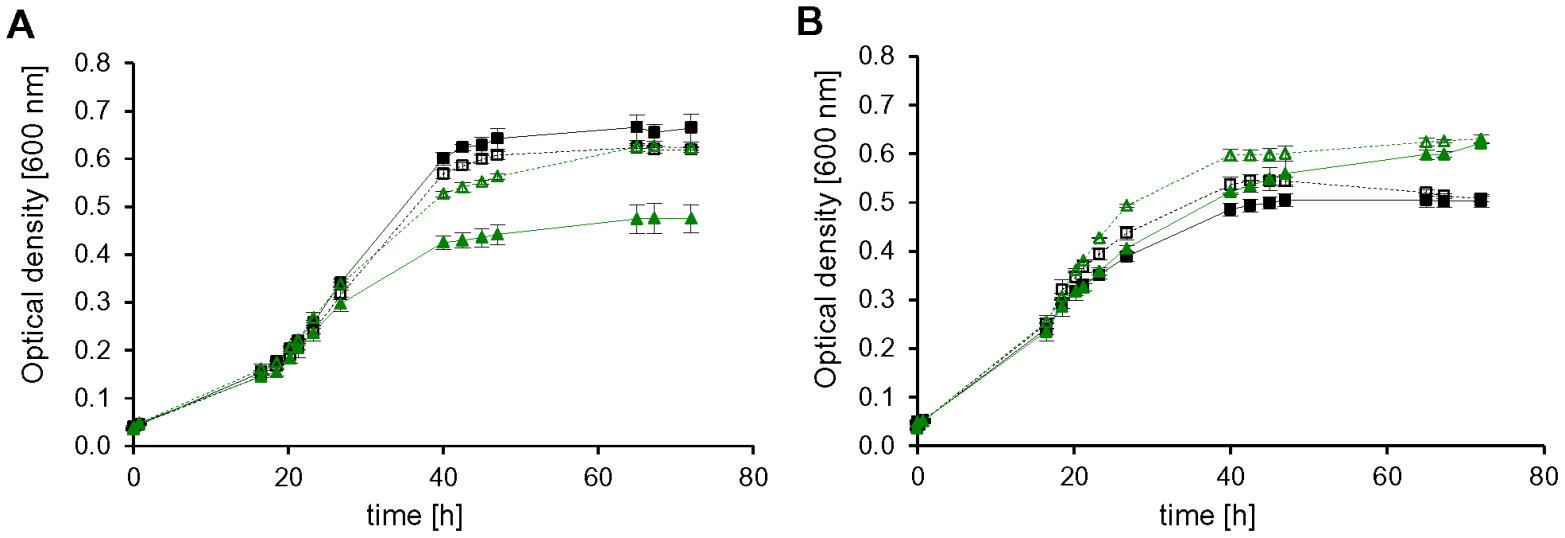
Growth of *aIF2γ* depletion mutant on two different carbon sources. Growth of *aIF2γ* depletion mutant (Depl_1901, green lines) and the wild-type (black lines) in synthetic medium with glucose (A) and CAS (B) as carbon source, respectively. Strains were grown in medium with 100 µg/ml tryptophan (filled symbols, solid lines) or without tryptophan (empty symbols, dotted lines). Average results from triplicate cultures and their standard deviations are shown. The wild-type is shown as black squares, the depletion mutant of *aIF2γ* as green triangles.

Very unexpectedly, growth of the other four mutants was identical or very similar in the absence and in the presence of tryptophan, at least under most conditions. Growth of mutant *HVO_0117* encoding aIF6 was clearly influenced by tryptophan during growth on glycerol (Fig. 7D, dotted yellow line versus solid yellow line), but hardly influenced by tryptophan at the remaining five conditions. Growth of mutant *HVO_2300* encoding aIF5A was somewhat tryptophan-dependent during growth on glucose and pyruvate (Fig. 7A and B, dotted red line versus solid red line), but not under the other conditions. Mutant *HVO_1963* encoding aIF5B was undistinguishable from the wild-type under all conditions, irrespective of the presence or absence of tryptophan. Most unexpected was mutant *HVO_1901* encoding aIF2γ, which grew better in the absence than in the presence of tryptophan on glucose and CAS (Fig. 8A and B, dotted green versus solid green line). Possible explanations for these unexpected results for four of the five depletion mutants are discussed below.

## Discussion

### Essential and non-essential initiation factor genes of *H. volcanii*

Translation initiation is a key process in cellular physiology and thus translation initiation factors can be expected to be essential. In accordance with this view, 13 of the 14 eukaryotic orthologs of the haloarchaeal genes analyzed in this study are essential in yeast. Therefore, it was a surprise that 9 of these 14 genes could be successfully deleted in *H. volcanii*. The explanation why single gene deletions were possible is very clear-cut for 4 of the 9 genes. The factors aIF1A and aIF2β are redundantly encoded in *H. volcanii* and in both cases the second gene can take over after deletion of one of the two orthologs. The two aIF1A proteins contain 67% identical and 88% similar amino acids, thus it is not astonishing that they are functionally redundant (alignment see Fig. S5). The two aIF2β proteins are more diverse. Their size is considerably different, HVO_1678 is 135 amino acids long, while HVO_2242 contains a C-terminal extension and is 202 amino acids long (alignment see Fig. S4). In the common region they have an identity of 39% and a similarity of 72%. Nevertheless, it has been verified that the two genes are not independent from one another as a translatome analysis revealed a tenfold increased translational efficiency of the remaining gene (*HVO_1678*) in the mutant Δ*HVO_2242*. Interestingly in both cases the single gene deletion mutants exhibited phenotypic differences from the wild-type under some of the tested conditions, indicating that the two aIF1 proteins and aIF2β proteins have largely overlapping but not identical functions.

The *H. volcanii* genome also contains two genes that are annotated to encode orthologs of aIF1. However, *HVO_1946* turned out to be essential, while deletion of *HVO_B0284* did not lead to any phenotype under any of the tested conditions. The two encoded gene products have only an identity of 49% (alignment see Fig. S5), therefore, most probably *HVO_1946* encodes a *bona fide* aIF1 while the annotation of *HVO_B0284* is not correct and it encodes a protein probably not involved in translation initiation.

Deletion of *HVO_1934* and *HVO_2706*, which are annotated to encode two proteins with similarities to subunits of eIF2B, did also not result in a phenotypic difference to the wild-type under any of the tested conditions. Eukaryotic eIF2B is a GDP/GTP exchange factor that is important for the function of eIF2. However, for aIF2 from *Sulfolobus* it has been shown that it binds GDP and GTP with the same affinity [34]. If that would also be true for *H. volcanii*, an exchange factor would not be required. In addition, eukaryotic eIF2B is a heteropentamer and thus it does seem likely that homologues of two of the five subunits could have the full function in archaea. On the other hand, it has been shown for an aIF2B subunit from *Pyrococcus horikoshii*, *P. furiosus* and *Thermococcus acidophilum*, respectively, that it binds to the cognate aIF2α subunit *in vitro* [39]. In addition, affinity purification of aIF2B from *T. kodakaraensis* led to the co-purification of aIF2α, among other proteins. Therefore, at the moment it is open whether *HVO_1934* and *HVO_2706* are initiation factors or whether the annotation is misleading and the encoded proteins have other functions.

It is also not clear whether the gene product of *HVO_1333* is involved in translation initiation. It belongs to the family of DEAD box helicases and has similarities to eIF4A. However, eIF4A forms a complex with eIF4E and eIF4G, which are not present in archaea. Therefore, it remains to be clarified whether this “aIF4A” is really involved in translation initiation and, if so, how it is recruited to the preinitiation complex. The gene is present in most or all archaea, indicating an important function, but the deletion mutant had a small phenotype only under two conditions.

The most surprising result was that *HVO_0699* could be deleted, as it encodes the α subunit of aIF2. As aIF2 is involved in guiding the initiator tRNA to the ribosome, its function is thought to be essential in archaea and eukaryotes. In addition, we have shown that the other two subunits, aIF2β and aIF2γ, are indeed essential in *H. volcanii* (further discussion of aIF2 see below).

Five of the 14 genes encoding aIF1, aIF2γ, aIF5A, aIF5B and aIF6 were found to be essential. Table 4 gives an overview of the 14 factors, their essentiality in *H. volcanii* and in yeast, and the proposed function in the crenarchaeal species *S. solfataricus* and in eukaryotes. In summary, our comprehensive deletion analysis identified 10 genes encoding 8 subunits of 6 aIFs that are essential or very important (aIF2α). In addition, we have shown that the ribosome dissociation factor ABCE1, which is involved in ribosome recycling in archaea and eukaryotes [40], is also essential in *H. volcanii* (Hammelmann and Soppa, unpublished results). This study of the importance of aIFs in the euryarchaeon *H. volcanii* nicely complements the results gained with the crenarchaeon *S. solfataricus* over the years, most of which have been obtained *in vitro* [2], [7], [11]. They will guide future experiments aimed at the molecular characterization of the *in vivo* functions of haloarchaeal aIFs.

**Table 4:** Translation initiation factors of *H. volcanii* and their comparison to orthologs form *S. solfataricus* and eukaryotes.

Gene	Factor	Essential in *S.c.*	Homologues			Proposed function in	
			Bac.	*S.c.*	Euk.	*S. solfataricus*	Eukaryotes
**DISPENSABLE**							
*HVO_B0284*	aIF1	yes [68]	-	Sui1	eIF1	Binds to 30S subunit; stimulates binding tRNAi and mRNA to ribosome [29]	Promotes fidelity of initiation codon selection and ribosomal scanning; Stimulates binding of eIF2-GTP-Met-tRNAi to 40S subunit
*HVO_1934*	aIF2Bα	no [69]	-	Gcn3	eIF2Bα	not examined	Guanosine nucleotide exchange factor; promotes GDP-GTP exchange on eIF2
*HVO_2706*	aIF2Bδ	yes [69]	-	Gcd2	eIF2Bδ	not examined	Guanosine nucleotide exchange factor; promotes GDP-GTP exchange on eIF2
*HVO_0699*	aIF2α	yes [69]	-	Sui2	eIF2α	Stabilizes tRNAi binding [32]	Phosphorylation blocks GTP/GDP exchange [70]
*HVO_1333*	eIF4A homolog	yes [76] (d.m.)	-	Tif1/Tif2	eIF4A	No eIF-4A homolog found	DEAD-box ATPase and ATP-dependant RNA helicase
**REDUNDANTLY ENCODED BUT ESSENTIAL**							
*HVO_0136*	aIF1A-1	yes [67]	IF1	Tif11	eIF1A	Stimulates binding of aIF2 to 30S subunit (in conjunction with aIF1) [30]	Stimulates binding of eIF2-GTP-Met-tRNAi to 40S subunit; stimulates subunit joining
*HVO_A0637*	aIF1A-2	yes [67]	IF1	Tif11	eIF1A	Stimulates binding of aIF2 to 30S subunit (in conjunction with aIF1) [30]	Stimulates binding of eIF2-GTP-Met-tRNAi to 40S subunit; stimulates subunit joining
*HVO_1678*	aIF2β-1	yes [69]	-	Sui3	eIF2β	function elusive [32]	Includes domains for binding of eIF2B and eIF5 [45]
*HVO_2242*	aIF2β-2	yes [69]	-	Sui3	eIF2β	function elusive [32]	Includes domains for binding of eIF2B and eIF5 [45]
**ESSENTIAL**							
*HVO_1946*	aIF1	yes [68]	YciH (some phyla)	Sui1	eIF1	Binds to 30S subunit; stimulates binding tRNAi and mRNA to ribosome [29]	Promotes fidelity of initiation codon selection and ribosomal scanning; Stimulates binding of eIF2-GTP-Met-tRNAi to 40S subunit
*HVO_1901*	aIF2γ	yes [69]	-	Gcd11	eIF2g	Structural core of aIF2; contains G-domain; stimulates interaction of tRNAi with 30S subunit [32]	Binding of tRNAi; contains GDP/GTP binding domain [71]
*HVO_2300*	aIF5A	yes [72]	-	Domain found in IF2B/IF5	eIF5A	not examined	Stimulates GTPase activity of eIF2 [73]
*HVO_1963*	aIF5B	yes [74]	IF2	Fun12	eIF5B	Ribosome dependant GTPase; adapts tRNAi in ribosomal P site, enhances translation of leaderless & leadered mRNAs [57]	Ribosome dependant GTPase; mediates ribosomal subunit joining [75]
*HVO_0117*	aIF6	yes [63]	-	Tif6	eIF6	translational inhibitor; prevent ribosomal subunit association [64]	Ribosome anti-association factor [65]; control of ribosome synthesis [66]

*S.c.* = *Saccharomyces cerevisiae*; Bac. = bacteria; Euk. = eukaryotes; d.m. = double mutant.

### Unexpected phenotypes of depletion mutants of essential aIFs

Northern blot analyses revealed that the transcripts of all five essential *aIF* genes could be successfully depleted in the constructed depletion mutants. However, the time courses of transcript depletion after the removal of the inducer tryptophan were very different, indicating drastically different stabilities of *aIF* transcripts. A genome-wide analysis of transcript stabilities in *Halobacterium salinarum* had revealed that the average half life is around ten minutes [41]. Two of the analyzed transcripts had much shorter half lives, the transcripts of *HVO_1963* and *HVO_2300* were hardly detectable only 2 minutes after the removal of tryptophan. The half lifes of the *HVO_0117* and *HVO_1946* were found to be not very far from the average value. However, the *HVO_1901* transcript had a half life of 90 minutes and has thus a nearly tenfold higher stability than an average haloarchaeal transcript. These results indicate that aIF2γ is essential for translation initiation under all conditions and there is no need for its down-regulation. By the same argument, the results could indicate that there are environmental conditions that require a fast down-regulation of the levels of aIF5A and aIF5B, despite their essentiality under “normal” conditions used for mutant construction in this study.

The inability to detect a single deletion mutant in more than 100 analyzed clones in five cases is a very strong argument if not a proof that these five genes are indeed essential. In the course of the characterization of replication genes of *Halobacterium salinarum* a failure to detect a deletion mutant in only 40 characterized clones was taken as evidence that the respective gene is essential [42]. In addition, the five yeast orthologs are also essential, therefore the result was not unexpected. As mentioned above, faithful translation initiation is extremely important for the survival of species, and at the start point of the project a much higher fraction of essential genes had been expected, similar to other species like yeast (Table 4).

However, it was totally unexpected that in the absence of tryptophan only one depletion mutant had a severe growth defect under all tested conditions, while the other four mutants exhibited phenotypes only under one or a few conditions or even grew indistinguishably from the wild-type under the six conditions tested. In addition, it was unexpected that the phenotypic differences in the presence and absence of tryptophan were rather low or even totally absent in four of five cases (Fig. 7, 8, and S3).

It should be noted that this counter-intuitive and at first sight contradictive lack of a severe phenotype after depletion of proteins encoded by genes that cannot be deleted was observed not only for aIFs and not only by us. The same observation was made for the ribosome dissociation factor ABCE1 (Hammelmann and Soppa, unpublished results) as well as for proteins involved in replication (Stewart McNeill, University of St. Andrews, UK, personal communication) and in recombination (Thorsten Allers, University of Nottingham, UK, personal communication) in *H. volcanii*. Therefore, it seems that conditional depletion of various essential gene products involved in several different biological processes does not lead to cell death in *H. volcanii* at least under the commonly applied laboratory conditions. The qualitative and fundamental difference between gene deletion and depletion mutants is that the level of the gene product is zero in the former, while a small amount of the gene product will be present in the latter. It is well known that the activity of prokaryotic promoters is never zero in a very strict sense, even in the absence of an inducer. Therefore, the transcript amount is not zero in depletion mutants, even if a Northern blot analysis might not be sensitive enough to visualize it. In addition, the discrepancy in the protein level could be lower than the discrepancy in the transcript level if translational regulation would occur. It was indeed shown that differential translational regulation is rather common in haloarchaea and growth phase-dependent translational regulation occurs in about 6% of all transcript of *H. volcanii* [43]. In most cases the regulatory effect was not larger than twofold, but deletion of *HVO_2242* has revealed that a tenfold up-regulation of translational efficiency of the transcript of the orthologous gene *HVO_1678* is possible in *H. volcanii*. However, deletion of a gene is a rather artificial situation, and in addition, the transcript level differences after depletion were larger than tenfold, therefore it seems very unlikely that the protein levels remained unchanged after transcript depletion. It rather seems that depletion of the protein levels of four of the five essential aIFs did not result in a visible phenotype under some or even most of the tested conditions. The results show that the importance of aIFs is different under various conditions, e.g. depletion of aIF6 has a very severe effect during growth on glycerol, but not at any of the other five conditions, or depletion of aIF5A resulted in a considerable growth defect during growth on pyruvate, but not under other conditions (Fig. 8). Therefore, the results exemplify that experimental conditions in the laboratory only very marginally mirror the life of microorganisms in real ecosystems, and that as many different conditions as possible should be tested to get an impression of the *in vivo* importance of gene products. In addition, the results also exemplify that a too high concentration of a protein can be of disadvantage. The depletion mutant of aIF2γ grows indistinguishably from the wild-type in the absence of tryptophan, but has a considerable growth defect when *HVO_1946* expression is induced (Fig. 8 and Table 3). In summary, the results revealed that depletion of an essential aIF can result in a severe growth defect under all tested conditions (aIF1) or only a single condition (aIF6), and counter-intuitively, that a too high concentration of an aIF can also inhibit growth (aIF2γ, Fig. 8).

### Similarities and differences of the initiator tRNA-binding factors aIF2 and eIF2

In both archaea and eukaryotes the heterotrimeric initiation factor a/eIF2 is comprised of the three subunits α, β and γ, which are all orthologs between the two domains. In both domains, a/eIF2 is involved in recruiting the initiator tRNA to the ribosome and thus a/eIF2 can be predicted to have fulfilled this function in the common ancestor of archaea and eukaryotes. It could even be shown that aIF2 can substitute for eIF2 in early steps of initiation, including cap-recognition and scanning to the correct start codon [44]. However, aIF2 is unable to replace eIF2 in later steps like subunit joining. Therefore, aIF2 inhibits instead of promoting translation in a mammalian *in vitro* translation system. Hence, besides the high degree of conserved function marked differences exist.

Eukaryotic eIF2 binds tRNA and GTP, brings the initiator tRNA to the small ribosomal subunit and requires GTP hydrolysis to leave the ribosome. It binds the initiation tRNA via the two subunits eIF2γ and eIF2β. The subunit eIF2α is not involved in tRNA binding and has regulatory functions. eIF2 binds GTP with higher affinity than GDP and requires two further eIFs for GTP hydrolysis and GDP/GTP exchange, namely eIF5 and eIF2B [40]. The interactions of both factors with eIF2 are mediated by the β subunit.

In contrast, the archaeal aIF2 from *S. solfataricus* binds GDP and GTP with equal affinity, and thus a GDP/GTP exchange factor is not required for aIF2 function [34]. In addition, aIF2 from *Sulfolobus* binds first to the ribosome and then attracts initiator tRNA [2]. Furthermore, the structures of aIF2 from *S. solfataricus* revealed that in contrast to eukaryotic eIF2 the archaeal aIF2 binds the initiator tRNA via the two subunits aIF2α and aIF2γ, while aIF2β does not have any contact to the tRNA.

In view of the structural and biochemical results obtained with the *Sulfolobus* aIF2 it was very surprising that the gene for the α subunit of the *H. volcanii* aIF2 could be deleted, because a subunit with extensive contacts to the initiator tRNA should be essential. One possible explanation could be that haloarchaeal aIF2 binds the initiator tRNA via the β and γ subunits, like in eukaryotes, and that the properties of the crenarchaeal aIF2 from *S solfataricus* cannot be generalized to all archaea. Crenarchaeota and euryarchaeota are very different in several respects, e.g. in the ploidy level or the cell division mechanism [45], [46].

In *Sulfolobus*, the γ subunit is at least threefold more abundant than the α and β subunits. It has been found that the γ has an additional role different from translation initiation. It can bind to the 5′-ends of transcripts provided that these carry a triphosphate group and it was shown that this binding can protect mRNA from degradation *in vivo* [7], [33]. These data indicate that aIF2γ is a dual function protein that links translation with RNA metabolism. It remains to be clarified whether aIF2γ has the same dual function role in *H. volcanii* or generally in euryarchaeotes. The growth defect after overexpression of aIF2γ could be a first indication that this is indeed the case. In haloarchaea the majority of transcripts are leaderless and thus a 5′-end binding protein would interfere with translation initiation. Therefore, the γ subunit could be essential for translation initiation in the context of the heterotrimeric initiation factor aIF2, while, in contrast, the γ subunit alone could be an inhibitor of translation initiation. Further analyses are required to analyze this theory. Nevertheless, the deletion and depletion experiments have revealed that both the presence of a basal level as well as a not too high intracellular concentration of aIF2γ are important in *H. volcanii*.

### Conclusions and Outlook

For the first time a comprehensive characterization of the *in vivo* importance of all annotated translation initiation factors in archaea has been performed. An astonishingly large number of translation initiation factor genes, 9 out of 14, could successfully be deleted. In two cases it was shown that this was due to the presence of two orthologous genes, which cannot be deleted simultaneously, and that the functions of aIF1A and aIF2β are also essential. Surprisingly, the gene encoding the α subunit of the heterotrimeric factor aIF2 could be deleted, which was unexpected because aIF2α of *Sulfolobus* is involved in initiator tRNA binding. However, the deletion mutant had a severe growth defect under all tested conditions. Therefore, 10 of the 14 genes encode essential or very important aIFs. Conditional depletion mutants of the five essentials genes were constructed and transcript depletion was verified. Surprisingly, only in one example depletion resulted in a severe growth defect under all conditions, while other examples exhibited a growth defect only under one or a few conditions and in one case the depletion was advantageous for *H. volcanii* at least at one condition. Comparison of the results obtained in this study with *H. volcanii* with the known function of eukaryotic eIFs indicate that homologous proteins do not necessarily need to have the same function in the two domains of life. Notably, the two domains of life use mutually exclusive mechanisms for translation initiation and thus common primordial functions as well as derived different function of essential factors are possible. The complete set of deletion and depletion mutants of haloarchaeal initiation factor genes opens the possibility for a future detailed analysis of their molecular biological functions and interactions.

## Materials and Methods

### Archaeal strains, media and growth conditions

*H. volcanii* strain H26 was obtained from Thorsten Allers (University of Nottingham, UK) [35]. This strain contains a *pyrE2* deletion and is thus auxotrophic for uracil, which is an important feature for the construction of deletion and depletion mutants (see below).

Cultures were grown aerobically in 2.1 M NaCl at 42°C as described [47] with various C sources. Complex medium contained 0.3% (w/v) yeast extract and 0.5% tryptone (w/v) and synthetic media contained, respectively, 0.25% (w/v) casamino acids (CAS), 0.5% (w/v) glucose, 0.5% (w/v) sucrose, 20 mM sodium pyruvate or 40 mM sodium acetate. Variations in salt concentrations and temperatures are described in Results and Discussion.

### Construction of deletion mutants

Gene deletion mutants were constructed using the Pop-In-Pop-Out method as described previously [35], [36]. For each gene two PCR fragments of about 500–600 nt were amplified that represented the upstream region with the 5′-end of the gene and the 3′-end of the gene and the downstream region, respectively. Primer sequences are available upon request. The two fragments were fused via a consecutive PCR reaction, resulting in a fragment containing an in frame deletion version of the gene and upstream and downstream regions. This fragment was cloned into the vector pMH101 using restriction selection cloning [36]. The sequence of the cloned region was verified and the plasmid was used to transform *H. volcanii Δdhfr*. Pop-In clones were selected in synthetic medium in the absence of uracil. Subsequently Pop-Out clones were selected in the presence of uracil and 5-fluoro orotic acid (5-FOA). The genomic organizations of Pop-In and Pop-Out clones and wild-type were analyzed using PCR reactions and Southern blot analysis. If no deletion variants were found among the Pop-Out clones, at least 100 clones were analyzed before it was concluded that the respective gene is essential.

### Construction of depletion mutants

For construction of the depletion mutants the plasmid pTA131 was used [35]. To enable the conditional control of gene expression the promoter of the tryptophanase gene (*HVO_0009*) was amplified and fused to the 5′-half of the open reading frame of the respective gene. Primer sequences are available upon request. The P_tna_ promoter is tightly controlled by tryptophan and was successfully used for the depletion of the essential gene *cct1* from *H. volcanii* [37]. The sequence of the cloned region was verified and the respective plasmid was used to transform *H. volcanii* H26. Clones that had integrated the plasmid via homologous recombination with the respective genomic gene copy were selected by growth in synthetic medium in the absence of uracil. This recombination placed an intact copy of the gene under the control of the tryptophanase promoter and thus the selection was performed in the presence of tryptophan. Genomic organization of wild-type and mutant was verified by Southern blot analysis. For conditional depletion of gene expression, cultures were grown to mid-exponential growth phase, split into half, and tryptophan was omitted from the depletion culture and added to the control culture.

### Growth in microtiter plates

For growth experiments in microtiter plates exponentially growing precultures were grown in Erlenmeyer flasks in complex medium. Cells were washed and inoculated as described by Jantzer *et al.* [38]. Various C sources, salt concentrations and temperatures were analyzed.

### Isolation of free and polysome-bound RNA

RNA isolation was performed as described by Lange *et al.* [19]. *H. volcanii* cultures were grown in 300 ml flasks to a cell density of 6×10^8^ cells/ml. After collection, cells were resuspended in buffer A and sonicated (3×30 s, output control 2, duty circle 50%, Branson Sonifier 250). A modification of sucrose gradients of 15% (w/v) to 45% (w/v) sucrose in buffer A was performed. RNA from sucrose gradients was isolated as described before [43].

### Northern blot analysis

RNA was isolated from exponentially growing cells as described by Chomczynski and Sacchi [48]. For transcript analysis of depletion mutants, cells were grown in synthetic medium with 100 µg/ml tryptophan. Cells of an exponentially growing preculture were centrifuged at 3320× g for 15 min and washed in 2.1 M salt solution. Cell pellets were resuspended in 1 ml 2.1 M salt solution and inoculated in fresh synthetic medium with or without tryptophan. RNA was isolated directly after inoculation as well as 15 minutes, 45 minutes and 90 minutes afterwards. From these RNA preparations, 3 µg were separated on a 1.8% agarose gel and transferred to nylon membranes. DNA-oligonucleotide probes complementary to the depleted genes were used as probes for hybridization.

### Quantitative real-time PCR

The RNA was isolated as described before. The DNase treatment, reverse transcription and real-time PCR were performed as described by Brenneis *et al.* [15].

The results for the real-time PCR were analyzed using the ΔC_t_ method including two normalization steps: one to the internal unregulated control *hpyA* (determined with the primers hpy_2f and hpy_2r) and the other one to one of the samples (determined with the primers qRT_1901_for and qRT_1901_rev). The C_t_ values of the control transcript *hpyA* were used to normalize the C_t_ levels of the *aIF2γ* transcripts.

### DNA microarray analysis

Free RNA as well as polysome-bound RNA was isolated as described above and reverse transcribed into Cy3- or Cy5-labeled cDNA using random hexamer primers and M-MLV reverse transcriptase RNase H minus (Promega, Mannheim, Germany). The DNA generation and preparation for hybridization on self-constructed DNA microarrays for *H. volcanii* was performed as described before [43]. Dye-swap experiments were included to exclude effects of disproportionate incorporation of Cy-3- or Cy-5-dUTP. To determine the influence of the aIF deletions, ribosome-bound RNA of the wild-type and the deletion mutant were compared. Some specific 60-mer oligonucleotides probes were added to the existing DNA microarray e.g. *HVO_1678*. Three independent experiments were performed and the data analyses of the results were performed as described in reference [43].

### Quantification of ATP levels

To measure the ATP level the cultures were grown in complex media to a cell density of 5–6×10^8^ cells/ml. 30 ml of the culture were pelleted (6000 rpm, 10 min, 20°C) and resuspended in the same volume of 2.1 M NaCl. For cell lysis 100 µl of this suspension were transferred to 5 ml ice-cold 10 mM sodium phosphate buffer (pH 7). For the measurement 500 µl cell extract was mixed with 25 µl of the luciferase reagent “Firefly lantern extract”. After vortexing the light emission was measured at 355 nm. For the generation of a standard curve ATP was dissolved in 10 mM sodium phosphate buffer and basal salt (50∶1), and amounts of 0 pmol ATP to 100 pmol ATP were mixed with 25 µl of the luciferase reagent “Firefly lantern extract” and light emission was quantified.

## Supporting Information

Figure S1 **Phenotypical characterization of all tested** ***aIF*** **deletion mutants in different media.** Nine gene deletion mutants and the *H26Δdhfr* wild-type were cultivated in microtiter plates on six different C-sources. The growth curves of the wild-type (filled black squares, dotted line) and all deletion mutants are shown in semi-logarithmic plots. Cultures were grown in complex medium (A) and synthetic medium with CAS (B), glucose (C), pyruvate (D), sucrose (E), acetate (F) as carbon source as well as 0.7 M NaCl (G) and 4 M NaCl (H). Average results from triplicate cultures and their standard deviations are shown. Identical color codes for the mutants of Figures 3 were used.
(TIF)

Figure S2 **Phenotypical characterization of all tested** ***aIF*** **deletion mutants at different temperatures.** Nine gene deletion mutants and the *H26Δdhfr* wild-type were cultivated in microtiter plates with complex media (A and C) and synthetic media with glucose as C-sources (B and D). Plates were cultivated at 30°C (A and B) and 50°C (C and D). Average results from triplicate cultures and their standard deviations are shown. Color codes are shown in Figure 3.
(TIF)

Figure S3 **Phenotypical characterization of all conditional depletion mutants.** Five gene depletion mutants and the *H26Δdhfr* wild-type were cultivated in synthetic medium with the 2.1 M NaCl and with glucose (A), pyruvate (B), sucrose (C), glycerol (D) and CAS (E) as carbon source. Figure F shows growth in synthetic medium with CAS at the elevated salt concentration of 4 M NaCl. Wild-type (black lines) and depletion mutants (colored lines) were cultivated in medium without tryptophan (empty symbols, dotted lines) or with 100 µg/ml tryptophan (filled symbols, continuous lines). Average results from triplicate cultures and their standard deviations are shown. The color code is given in Figure 7 and 8.
(TIF)

Figure S4 **Multiple Sequence Alignment of eIF2β subunit.** A multiple sequence alignment was constructed using Clustal Omega. The two protein sequences of *Haloferax volcanii* aIF2β subunit (*HVO_1678* and *HVO_2242*) are compared to the a/eIF2β sequences of different archaeal and eukaryotic organisms. Identical amino acids are tagged with stars. H.vol, *Haloferax volcanii*; M.maz, *Methanosarcina mazei*; S.sol, *Sulfolobus solfataricus*; S.cer, *Saccharomyces cerevisiae*; M.mus, *Mus musculus;* H.sap, *Homo sapiens*.
(TIF)

Figure S5 **Multiple Sequence Alignment of aIF1A and aIF1 translation initiation factors.** The multiple sequence alignment was constructed using Clustal Omega. The two protein sequences of *Haloferax volcanii* (A) aIF1A (*HVO_0136* and *HVO_A0637*) as well as (B) aIF1 (*HVO_1946* and *HVO_B0284*) are compared. Identical amino acids are tagged with stars.
(TIF)

Table S1 Translation initiation mechanisms.
(DOC)

Table S2 Growth of all single and double gene deletion mutants of dispensable and redundantly encoded genes for translation initiation factors of *H. volcanii*.
(XLS)

Table S3 Growth of all conditional depletion mutants of essential translation initiation factors of *H. volcanii* with (d) or without (nd) depletion.
(XLS)
