# Supplementary figures and images for: A Comprehensive Analysis of the Importance of Translation Initiation Factors for Haloferax volcanii Applying Deletion and Conditional Depletion Mutants
Source: PLoS One. 2013 Nov 14;8(11):e77188. doi: 10.1371/journal.pone.0077188 (PMC3828320; doi:10.1371/journal.pone.0077188)

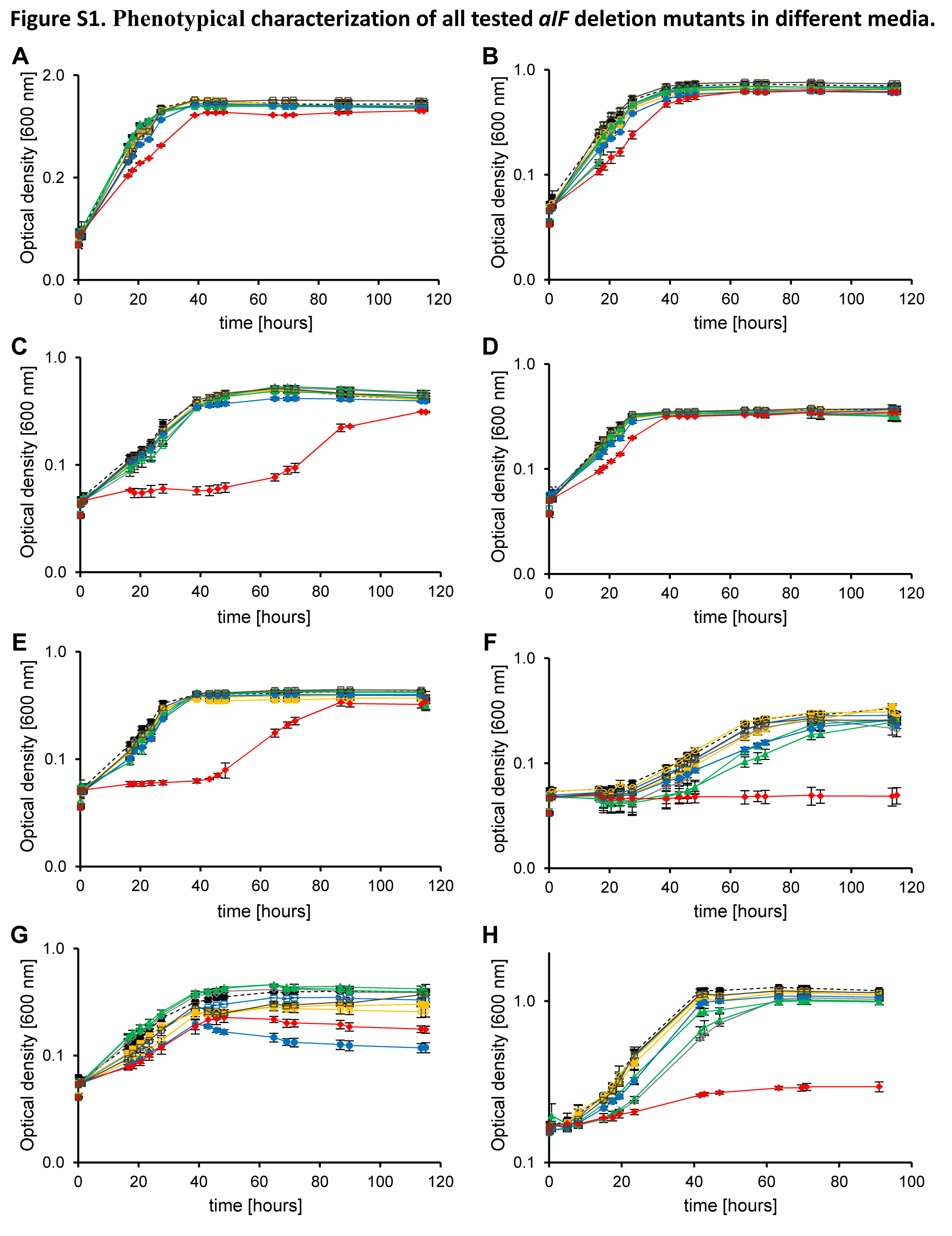

Supplement: Figure S1 — Phenotypical characterization of all tested aIF deletion mutants in different media. Nine gene deletion mutants and the H26Δdhfr wild-type were cultivated in microtiter plates on six different C-sources. The growth curves of the wild-type (filled black squares, dotted line) and all deletion mutants are shown in semi-logarithmic plots. Cultures were grown in complex medium (A) and synthetic medium with CAS (B), glucose (C), pyruvate (D), sucrose (E), acetate (F) as carbon source as well as 0.7 M NaCl (G) and 4 M NaCl (H). Average results from triplicate cultures and their standard deviations are shown. Identical color codes for the mutants of Figures 3 were used. (TIF) [file pone.0077188.s001.tif]

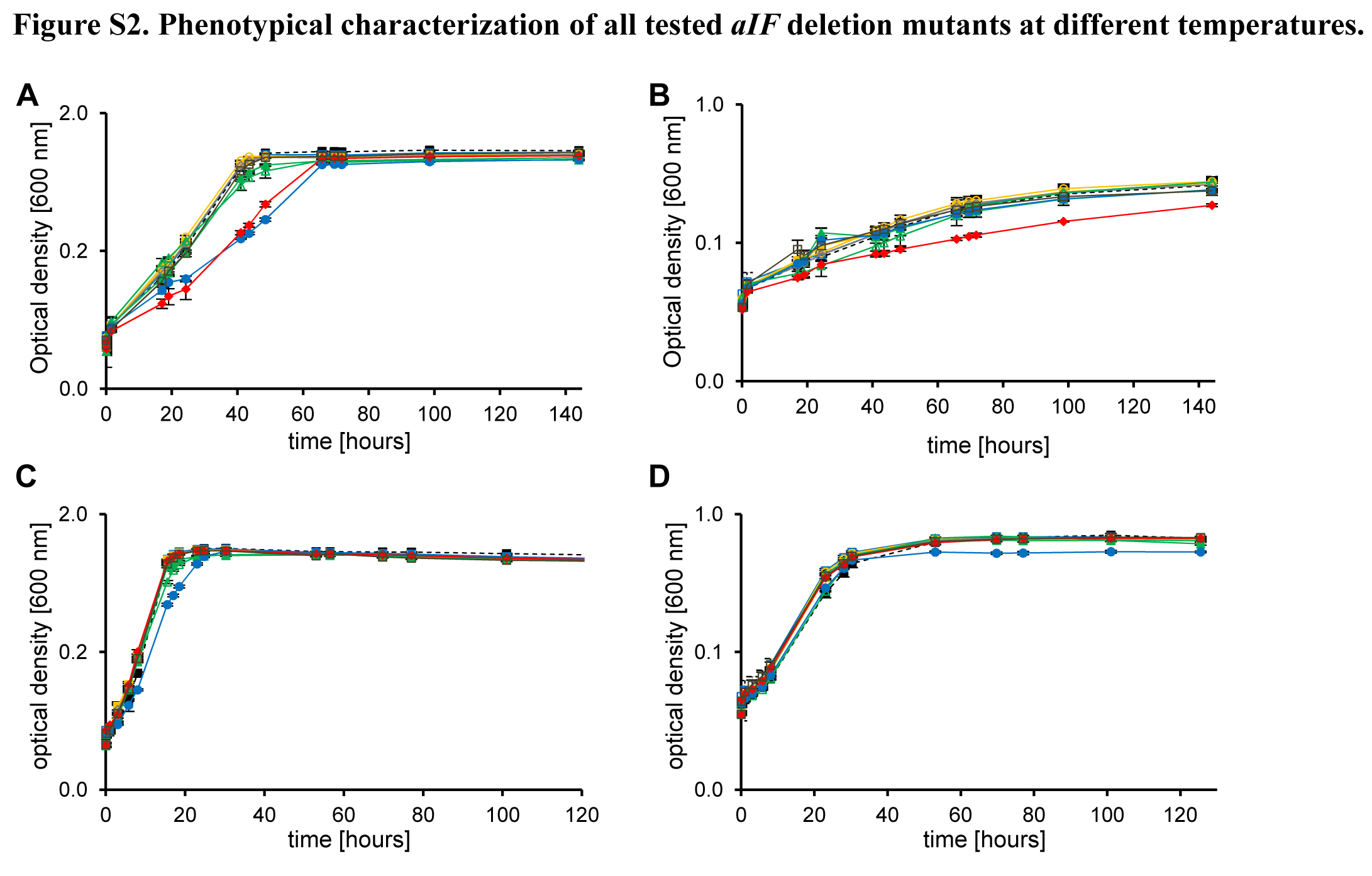

Supplement: Figure S2 — Phenotypical characterization of all tested aIF deletion mutants at different temperatures. Nine gene deletion mutants and the H26Δdhfr wild-type were cultivated in microtiter plates with complex media (A and C) and synthetic media with glucose as C-sources (B and D). Plates were cultivated at 30°C (A and B) and 50°C (C and D). Average results from triplicate cultures and their standard deviations are shown. Color codes are shown in Figure 3. (TIF) [file pone.0077188.s002.tif]

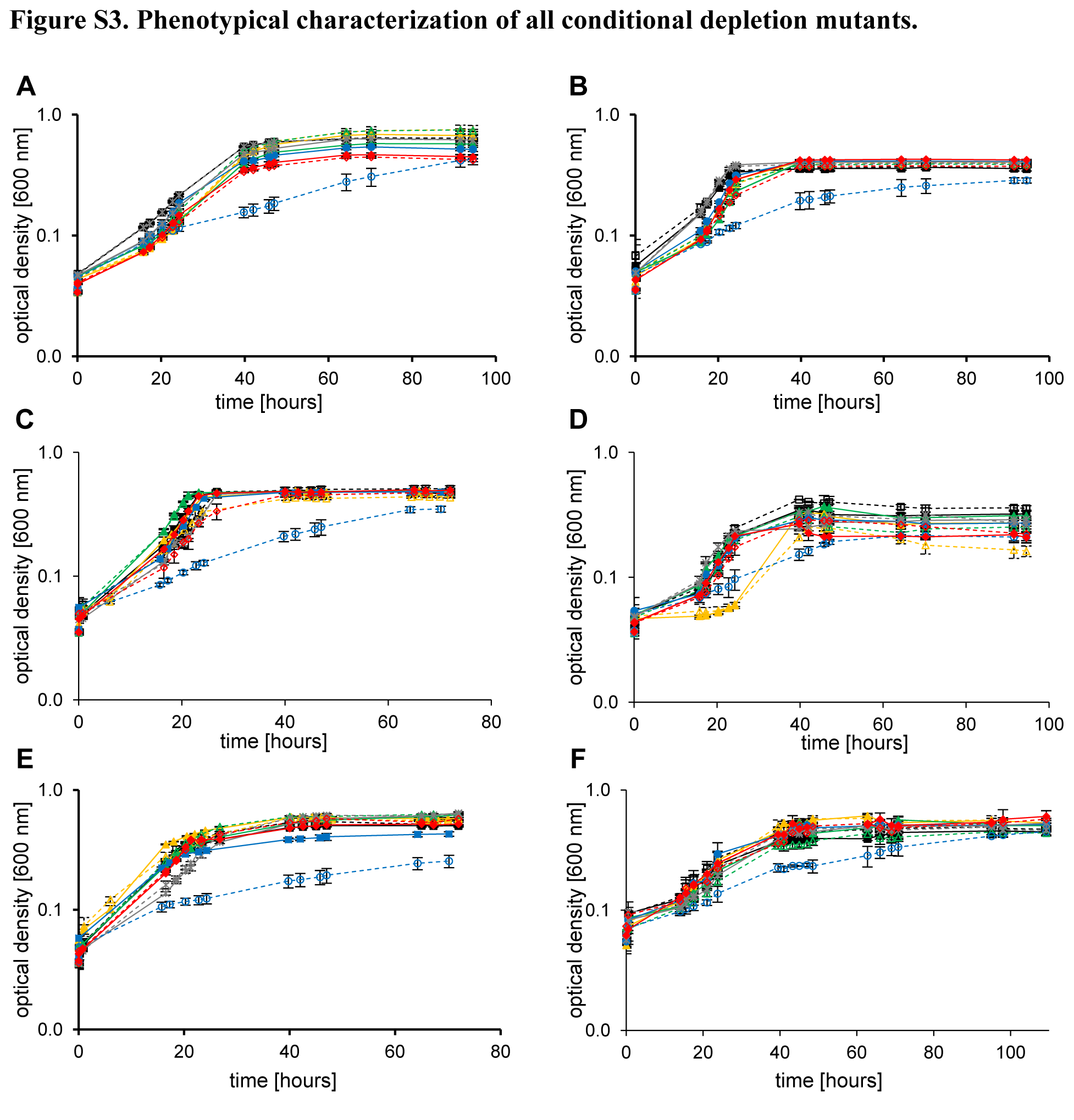

Supplement: Figure S3 — Phenotypical characterization of all conditional depletion mutants. Five gene depletion mutants and the H26Δdhfr wild-type were cultivated in synthetic medium with the 2.1 M NaCl and with glucose (A), pyruvate (B), sucrose (C), glycerol (D) and CAS (E) as carbon source. Figure F shows growth in synthetic medium with CAS at the elevated salt concentration of 4 M NaCl. Wild-type (black lines) and depletion mutants (colored lines) were cultivated in medium without tryptophan (empty symbols, dotted lines) or with 100 µg/ml tryptophan (filled symbols, continuous lines). Average results from triplicate cultures and their standard deviations are shown. The color code is given in Figure 7 and 8. (TIF) [file pone.0077188.s003.tif]

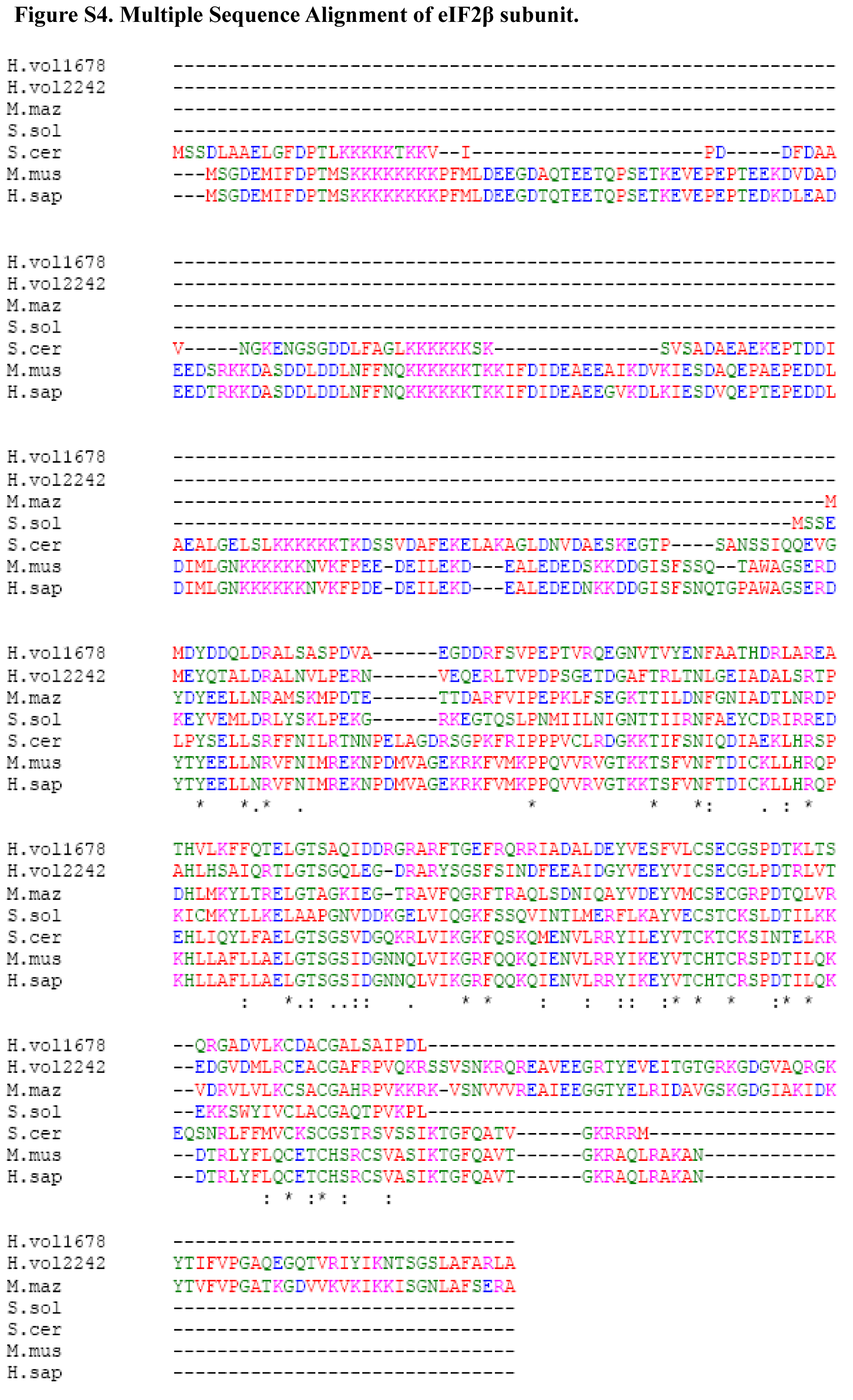

Supplement: Figure S4 — Multiple Sequence Alignment of eIF2β subunit. A multiple sequence alignment was constructed using Clustal Omega. The two protein sequences of Haloferax volcanii aIF2β subunit (HVO_1678 and HVO_2242) are compared to the a/eIF2β sequences of different archaeal and eukaryotic organisms. Identical amino acids are tagged with stars. H.vol, Haloferax volcanii; M.maz, Methanosarcina mazei; S.sol, Sulfolobus solfataricus; S.cer, Saccharomyces cerevisiae; M.mus, Mus musculus; H.sap, Homo sapiens. (TIF) [file pone.0077188.s004.tif]

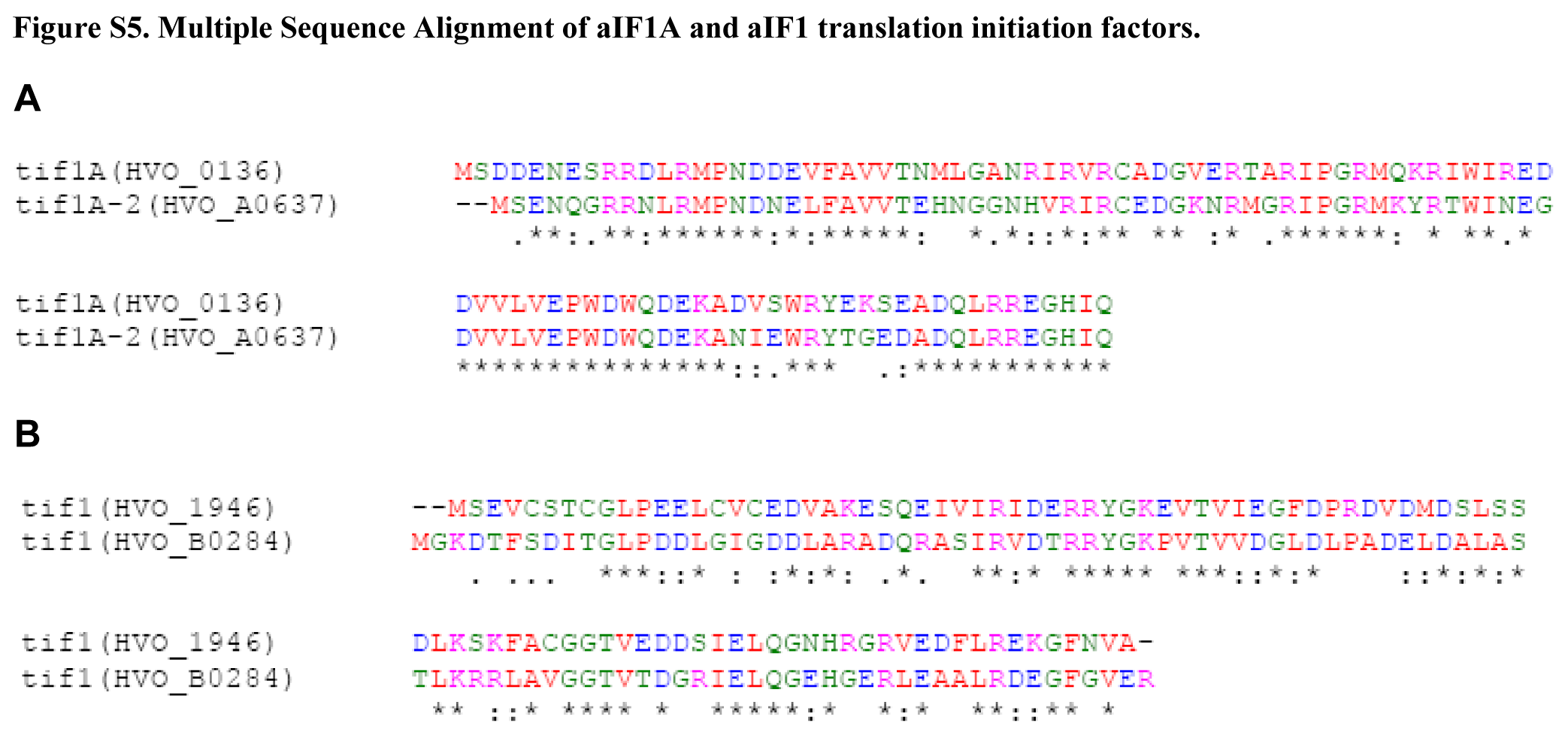

Supplement: Figure S5 — Multiple Sequence Alignment of aIF1A and aIF1 translation initiation factors. The multiple sequence alignment was constructed using Clustal Omega. The two protein sequences of Haloferax volcanii (A) aIF1A (HVO_0136 and HVO_A0637) as well as (B) aIF1 (HVO_1946 and HVO_B0284) are compared. Identical amino acids are tagged with stars. (TIF) [file pone.0077188.s005.tif]
