# Supplementary material for: A Comprehensive Analysis of the Importance of Translation Initiation Factors for Haloferax volcanii Applying Deletion and Conditional Depletion Mutants
Source: PLoS One. 2013 Nov 14;8(11):e77188. doi: 10.1371/journal.pone.0077188 (PMC3828320; doi:10.1371/journal.pone.0077188)
Supplement: Table S1 — Translation initiation mechanisms. (DOC) [file pone.0077188.s006.doc]

Table S1. Translation initiation mechanisms

| Mechanism | Domain | Characteristic features |
| --- | --- | --- |
| Shine-Dalgarno | Bacteria, Archaea | Base-pairing of SD sequence with 3’-end of 16S rRNA; fixed distance between SD and start codon; polycistronic mRNAs |
| Scanning | Eukaryotes | 5’-cap recognition by eIFs and recruitment of 40S subunit; linear scanning of 5’-UTR until first AUG is reached |
| IRES | Eukaryotes | Complex RNA structure within the 5’-UTR; recognition by ITAFs that recruit 40S subunit |
| Leaderless | Archaea, Bacteria, Eukaryotes | Complete ribosome & tRNAi bind the 5’-AUG; restricted to AUG; differential IF-dependence & antibiotic sensitivity |
| SD-less 5’-UTR | Haloarchaea; other Archaea and Bacteria? | SD and IRES are absent from 5’-leader; not inhibited by mutants that block scanning; efficiency depends on sequence of 5’-UTR |
